# Use of the *p*-values as a size-dependent function to address practical differences when analyzing large datasets

**DOI:** 10.1038/s41598-021-00199-5

**Published:** 2021-10-22

**Authors:** Estibaliz Gómez-de-Mariscal, Vanesa Guerrero, Alexandra Sneider, Hasini Jayatilaka, Jude M. Phillip, Denis Wirtz, Arrate Muñoz-Barrutia

**Affiliations:** 1 Bioengineering and Aerospace Engineering Department, Universidad Carlos III de Madrid, 28911 Leganés, Spain; 2 Instituto de Investigación Sanitaria Gregorio Marañón, 28007 Madrid, Spain; 3 Statistics Department, Universidad Carlos III de Madrid, 28903 Getafe, Spain; 4 Department of Chemical and Biomolecular Engineering, Institute for Nanobiotechnology, The Johns Hopkins University, Baltimore, MD 21218 USA; 5 AtlasXomics Inc., New Haven, CT 06511 USA; 6 Department of Biomedical Engineering, The Johns Hopkins University, Baltimore, MD 21218 USA; 7 Department of Oncology, The Johns Hopkins University School of Medicine, Baltimore, MD 21205 USA

**Keywords:** Computational biology and bioinformatics, Computational models, Data mining, Data processing, Statistical methods

## Abstract

Biomedical research has come to rely on *p-values* as a deterministic measure for data-driven decision-making. In the largely extended null hypothesis significance testing for identifying statistically significant differences among groups of observations, a single *p-value* is computed from sample data. Then, it is routinely compared with a threshold, commonly set to 0.05, to assess the evidence against the hypothesis of having non-significant differences among groups, or the null hypothesis. Because the estimated *p-value* tends to decrease when the sample size is increased, applying this methodology to datasets with large sample sizes results in the rejection of the null hypothesis, making it not meaningful in this specific situation. We propose a new approach to detect differences based on the dependence of the *p-value* on the sample size. We introduce new descriptive parameters that overcome the effect of the size in the *p-value* interpretation in the framework of datasets with large sample sizes, reducing the uncertainty in the decision about the existence of biological differences between the compared experiments. The methodology enables the graphical and quantitative characterization of the differences between the compared experiments guiding the researchers in the decision process. An in-depth study of the methodology is carried out on simulated and experimental data. Code availability at https://github.com/BIIG-UC3M/pMoSS.

## Introduction

**Figure 1:**
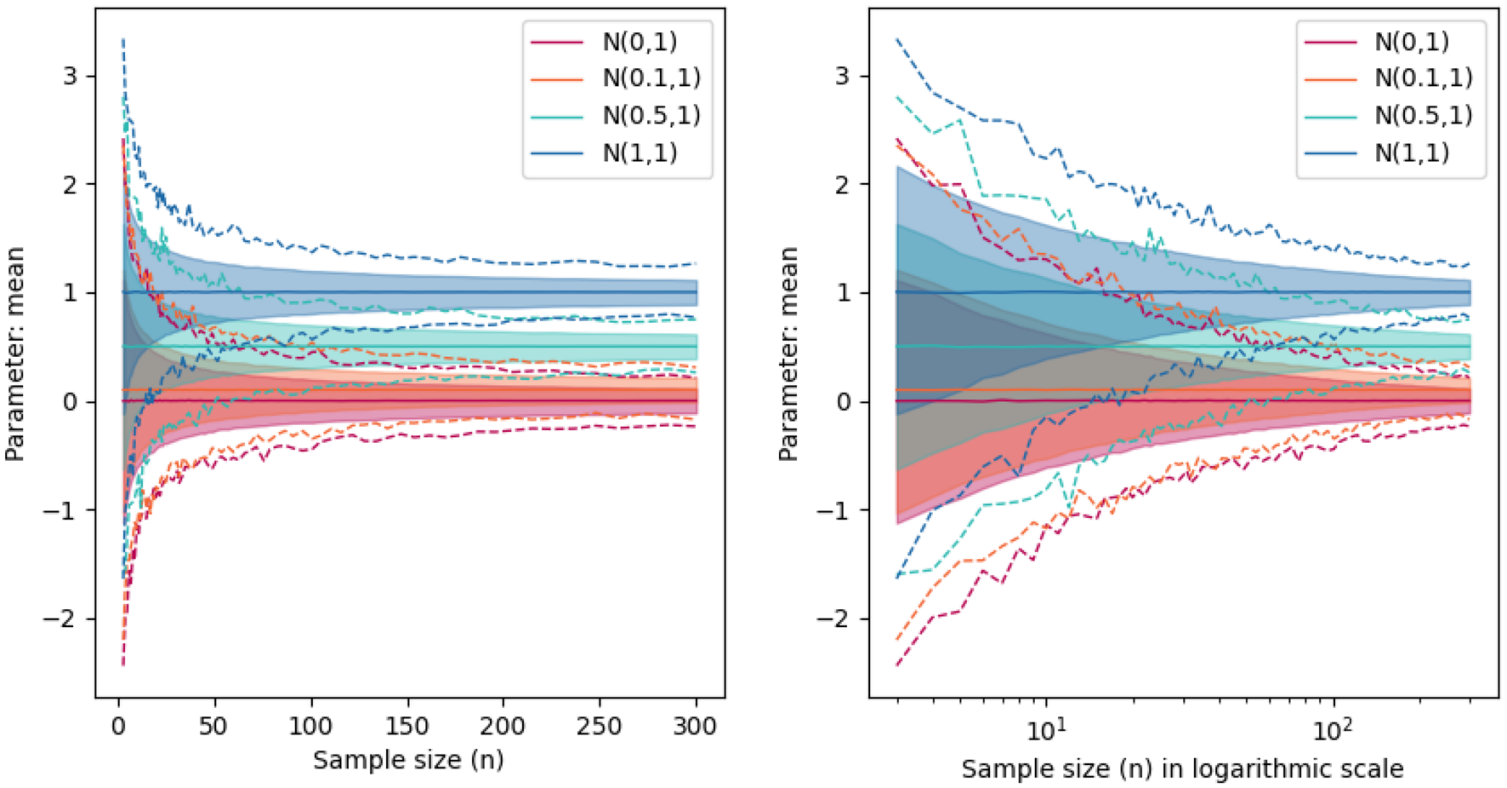
Bootstrapping estimation of two-sided $95\%$ confidence interval for the mean of different normal distributions with standard deviation of 1 and mean values of 0, 0.1, 0.5 or 1. For each value of the sample size, we compute the mean of a simulated normal distribution 15,000 times. The final confidence interval is obtained by clipping $95\%$ of the values among the 15,000 (filled area). The dashed lines show the maximum and minimum values of the sample mean value obtained for each sample size. The information is shown both in linear and logarithmic scale.

The ability to acquire, store and disseminate large amounts of data is constantly improving in life-science laboratories. Having such big datasets available for multiple kinds of analysis supports the proliferation of many different new methodologies for their study. Nonetheless, this data explosion has also exposed the challenges that classical statistical techniques need to face when analyzing such types of datasets. An extended practice in experimental life-science is the analysis of differences among experimental settings. In order to decide whether statistically significant differences exist, null hypothesis significance testing (NHST) is usually performed. Namely, a formal hypothesis test (e.g., Student’s t-test^1^) is stated in which the no effect hypothesis (e.g., the equality of the mean values yielded by experimental datasets), is assessed thanks to the computation of a *p-value* on sample data. This value is then compared with the threshold 0.05 to decide whether or not the null hypothesis is rejected. When working with datasets with large sample sizes, the accuracy of the estimators (mean values or other parameters) improves, Fig. 1 and Supplementary Fig. S1 in the Supplementary Information. While NHST nearly always finds statistical differences among the group means in datasets with large sample sizes, the researchers usually aim to find out whether those differences are *interesting*, e.g. biologically or clinically relevant. Technically, the *p-value* depends on the size of the data being tested: the larger the sample size, the smaller the *p-value*. An easy understanding of the latter relies on the evidence in the data against the null hypothesis instead of the existence of interesting differences among groups^2^. The larger the data size, the larger the accuracy of the statistical test, and therefore, the stronger the evidence against or in favor of the null hypothesis. The latter is in high contrast with the recurrent misleading interpretation of the *p-value* as a “gold standard” for the identification of biologically or clinically relevant differences among experiments^2–6^. In particular, when large sample sizes are available, life-scientists could detect statistically significant evidence against the null hypothesis through a small enough *p-value*, even though there are no interesting differences from the practical point of view. Even more, the *p-value* is itself a random variable that depends on the sample data used to estimate it^7,8^; and, therefore, it has a sampling distribution that is intrinsically determined by the noise in the data. The *p-value* is known to have a “wide sample to sample variability”^5^. A straightforward example is as follows: the *p-value* has a uniform distribution $\mathscr {U}(0,1)$ under the null hypothesis, which is rejected $5\%$ of the times when a significance threshold of $5\%$ is being used (Type I error). Hence, if many different samples were analyzed, in $5\%$ of the cases, a single computation of the *p-value* would lead to the wrong conclusion that there exist statistically significant differences among two groups identically distributed^9^. Similar to the examples in Refs.^3,5^, Supplementary Fig. S2 in the Supplementary Information further illustrates this behavior.

Notwithstanding, there remain many situations for which the ‘dichotomy’ associated with the *p-value* is necessary for data-driven decision-making^10^. Here, we present different approaches described in the literature to facilitate the interpretability of the *p-value*. First approach consists of Shannon information or *S-value* stated as:

1 $$\begin{aligned} s=-\log _{2}p, \end{aligned}$$

where *p* is the *p-value*)^2^. The *S-value* expresses the self-information given by the datasets with respect to the null-hypothesis rather than a probability or evidence against it. However, the *S-value*, same as the *p-value*, depends on the sample size and the datasets used to compute a single realization of them. Therefore, used as single numbers, they have a limited capacity to inform about practical relevance of the differences among the compared groups^2^. Other approaches analyze the distribution of empirically estimated *p-values*, also known as *p-curve*^11^, which does not take into account the effect of the sample size. Computing the *p-curve* for datasets with large sample sizes will result in a high frequency of *p-values* around zero regardless the differences among the compared groups. There are also works that focus on the sample-size-dependence and the sensitivity of the *p-value* to this size, commonly denoted by *n*^12^. The authors provide a detailed description of the drawbacks of NHST applied to large datasets and they suggest the use of confidence intervals (CI) and effect sizes as alternative measures. However, to the best of our knowledge, there are no methods that exploit the sample-size-dependence of the *p-value* to derive easily interpretable parameters to assess the existence of interesting differences from the biological or clinical perspective.

In this work, the authors propose to fit the relationship between the sample size and the *p-value* in the Mann–Whitney U-test to detect statistically significant differences among two or more observed groups of observations as an exponential function. The choice of an exponential model allows us to assess whether *bona fide* differences exist from the practitioner’s perspective rather than just statistically significant. Thus, this paper presents an easily interpretable tool to support biomedical researchers in their statistical analyses with large datasets.

The appropriateness of the exponential model is illustrated in “Materials” and the Supplementary Information. The choice of a Mann–Whitney U-test, instead of the Student’s t-test, to address the problem of finding evidence among the differences between groups is supported by its distribution-free assumptions. Then, for a given sample size *n* its *p-value*, *p*(*n*), is approximated using:

2 $$\begin{aligned} p(n) = a \cdot e^{-cn} \quad \text {where }\quad a, c \in \mathbb {R^{+}}. \end{aligned}$$

The values of *a* and *c* are found to minimize the squared differences between a set of *p-values* for many samples of size *n*, which are obtained using Monte Carlo cross-validation (MCCV)^13^, and its estimation using the exponential approach. The parameters *a* and *c* are associated with the dependency of the *p-value* on the sample size and quantitatively measure the relationship between the two or more groups being compared.

**Figure 2:**
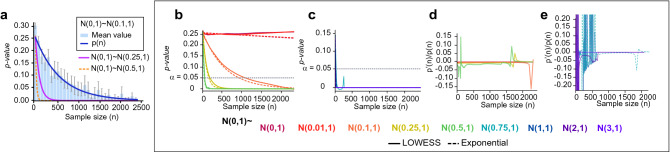
(**a**) The *p-value* is a random variable that depends on the sample size and can be modeled as an exponential function ($p(n) = ae^{-cn}$, Eq. 2). For each pair of normal distributions being compared, two subsets of size *n* are obtained by sampling from data generated following the corresponding normal distribution. Then, these datasets are compared using the Mann–Whitney statistical test and the *p-value* obtained is stored. The procedure is repeated many times for each size *n*. The blue bars with the standard error of the mean (SEM), show the distribution of all the *p-values* obtained at each size *n* when two normal distributions of mean 0 and 0.1, and standard deviation 1 are compared. The blue curve shows the corresponding exponential fit. The magenta and yellow curves represent the resulting *p*(*n*) function when a normal distribution of mean 0 and standard deviation 1 is compared with a normal distribution of the same standard deviation and mean 0.25 and 0.5, respectively. A normal distribution with a mean of 0 and a standard deviation of 1 is compared with a normal distribution of means 0, 0.01, 0.1, 0.25, 0.5, 0.75, 1, 2 and 3 respectively. Multiple *p-values* are calculated for sample sizes between 2 and 2500 (Supplementary Fig. S2 in the Supplementary Information). (**a**) and (**b**) Locally weighted scatter plot smoothing (LOWESS) fit to the mean *p-values* (red markers in Supplementary Fig. S2 in the Supplementary Information) computed for each value of the sample size *n*. Likewise, an exponential function is fitted to all the simulated *p-values*. (**b**) Comparison of $\mathscr {N}(0,1)$, with, $\mathscr {N}(0.01,1)$, $\mathscr {N}(0.1,1)$, $\mathscr {N}(0.25,1)$ and $\mathscr {N}(0.5,1)$. (**c**) $\mathscr {N}(0,1)$ is compared with $\mathscr {N}(0.75,1)$, $\mathscr {N}(1,1)$, $\mathscr {N}(2,1)$ and $\mathscr {N}(3,1)$. (**d**) and (**e**) Ratio between each LOWESS curve and its differential. Constant ratio and accurate exponential fits show empirically that the relationship between *n* and the *p-value* shows an exponential nature.

Note that *p*(*n*), Eq. (2), is used to compare pairs of experimental conditions; therefore, *p*(*n*) is computed as the exponential fit of the *p-values* computed on multiple samples of different sizes extracted from the large dataset at hand. Hence, the parameters *a* and *c* in Eq. (2) correspond to those defining the exponential fit. We use MCCV as the sampling strategy: for different values of the sample size *n*, two subsets of size *n* (one from each of the groups to be compared) are randomly sampled and compared with a statistical test. The resulting *p-value* is stored and the procedure is repeated many times, namely, many *p-values* are computed for each value of *n*. At the end of the procedure, a large set of *n*-dependent *p-values* is obtained. The MCCV process is then repeated for different values of *n* so the exponential function in Eq. (2) can be fitted (Fig. 2a). Although the compared sub-samples in MCCV have both sizes of value *n*, the original samples can have different sizes.

Similar to any exponential function, *p*(*n*) converges to zero whenever the distributions of the compared groups are not the same. On the contrary, if the distributions are equivalent, *p*(*n*) is constant as the *p-value* is uniformly distributed in such situation. The faster the function converges, the stronger is the evidence against the null hypothesis. In other words, a fast decay implies finding statistically significant differences between the groups at small sample sizes, i.e. differences appear early. In our simulation study, when normal distributions of standard deviation one and mean value in the range [0, 3] are compared, we see that the higher the difference among the mean values of each normal distribution, the faster the decay of the exponential function *p*(*n*), as expected (Fig. 2a). We will observe that the parameters *a* and *c* (Eq. 2) increase proportionally with the mean value of the distribution compared with $\mathscr {N}(0,1)$. Thus, *a* and *c* enable the spatial representation of each normal distribution with respect to $\mathscr {N}(0,1)$. These parameters can simplify the identification of the existence of interesting biological differences. Indeed, they can measure how far from each other the distributions of the groups being compared are.

With this new idea in mind, a robust decision binary index, $\theta _{\alpha , \gamma }$, can be mathematically defined (Eq. 10 in “Materials”) which depends on the significance level $\alpha$ and a regularization parameter $\gamma$ related to the convergence to zero of the exponential fitted function.

The idea behind the index $\theta _{\alpha , \gamma }$ is to gather the information about the *p-values* for different sample sizes against the predefined significance level $\alpha$, usually equal to $5\%$. A distance $\delta _{\alpha , \gamma }$ (Eq. 6 in “Materials”) is defined to compare the value of the function *p*(*n*) with $\alpha$ for each *n* value. The distance $\delta _{\alpha , \gamma }$ measures the difference between the areas under the constant function at level $\alpha$ and the area under the curve *p*(*n*). The distance $\delta _{\alpha , \gamma }$ is then used to obtain the binary index $\theta _{\alpha , \gamma }$ that indicates whether *p*(*n*) and the $\alpha$ constant are far from each other or not. If for most values of *n* the function *p*(*n*) is smaller than $\alpha$, then $\theta _{\alpha , \gamma } = 1$, which means that there are interesting differences among the datasets being tested. Otherwise, $\theta _{\alpha , \gamma } = 0$, which is interpreted as the non-rejection of the null hypothesis, and thus, the compared experimental set ups behave in a similar way.

As the exponential function is defined for all values $n\in (-\infty , +\infty )$, it is necessary to determine a range of *n* for which the function *p*(*n*) is meaningful in the context of this study. The decay of *p*(*n*) is concentrated in a range between $n=0$ and a certain value of *n* for which $p(n) \sim 0$ (convergence of *p*(*n*)); so, $\delta _{\alpha , \gamma }$ should be only calculated in that range. A parameter $\gamma$ is used as a regularizer to measure the sample size of convergence $n = n_{\gamma }$, such that $p(n = n_{\gamma }) \sim 0$. Small $\gamma$ values imply less restrictive decisions, i.e. $\theta _{\alpha , \gamma } = 1$ when the groups being compared do not show clear differences. Nonetheless, the experimental evaluation of the method over synthetic and real data evidences $\gamma = 5e^{-06}$ to be a reasonable choice (“Materials” and Supplementary Information). Note that when *p*(*n*) is determined simply by the definition of the parameters *a* and *c* in Eq. (2), the minimum sample size needed to observe statistically significant differences at $\alpha$-level can also be provided. As *p*(*n*) continuously decreases, the value of *n* for which *p*(*n*) is always smaller than $\alpha$ can be easily calculated. This value is called $n_{\alpha }$.

## Methods

Here, we first show the relation between the *p-value* and the size of the data being analyzed. Then, we justify the choice of an exponential model to fit the *p-value* as a function of the sample size *n*. With this idea in mind, we propose a methodology which considers the *p-value* as a function of the sample size *n* and determines when a statement of interesting differences can be made ($\theta _{\alpha , \gamma } = 1$ in Eq. 10). Once the problem is described technically, it is possible to calculate the minimum size $n_{\alpha }$ at which the null hypothesis of the test is statistically significant (Eq. 12). This parameter $n_{\alpha }$ can be used to characterize the data. Finally, the reliability of our method is rigorously tested.

### The effect of the data size in empirical estimators

Figure 1 illustrates that the precision on the mean estimators increases with the size of the data^12,14^. For each sample size, we use MCCV with simulated samples from normal distributions to calculate both the mean and its two sided $95\%$ (CI). Figure 1 does also display the maximum and minimum simulated values of the sample mean value for each sample size. In all cases, an exponential convergence of the CI and the maximum and minimum mean values to the estimated value is observed.

### *p-values* as an exponential model of data sizes

The Student’s t-test assumes that the datasets being compared follow a normal distribution, so to cover broader applicability, we choose the Mann–Whitney U test to develop the proposed methodology. We point out that this test also allows us to compare heteroscedastic groups. Nevertheless, the same steps can be applied using the Student’s t-test if the datasets meet the specific assumptions made in the test. Figure 2a and Supplementary Fig. S2 in the Supplementary Information illustrate the idea that the *p-value* in Mann–Whitney U-test is a function that depends on the sample size. In both figures, different randomly generated normal distributions are compared using the Mann–Whitney U statistical test^15^ to illustrate that there exists a continuous inverse relation between *p-values* and *n*, i.e. *p-values* decrease when *n* increases^3,14,16^. Figure 2a shows the decay of the mean *p-value* for each sample size *n* and its exponential shape. Either with Mann–Whitney U test^15^ or with Student’s t-test^1^, it can be proved that the obtained *p-value* converges to zero when the sample size is large and the distributions being assessed are not exactly the same, i.e., the *p-value* tends to zero when the sample size tends to infinity. A mathematical demonstration of this statement is available in the Supplementary Information.

Going a step further, we claim that the *p-values* can be indeed written directly as a function of *n*, *p*(*n*), and that this function adjusts well to an exponential function (see Fig. 2a). It is well known that the *p-value* associated with the *t*-statistic of the Student’s t-test has an exponential decay due to its direct relation with the *erf*(*z*) function^17^. Hence, to show this for the Mann–Whitney U-test we first estimate the value that the *p-value* function has at each possible value of *n*. This can be done easily with the MCCV^13^ by solving the test with many different samples of a given size *n*: at each iteration *i* of the procedure, $n = n_i$ is fixed, and two populations of size $n_i$ are compared. This procedure is repeated many times in each iteration *i* to cover the variability of the problem at $n=n_i$. At the end, we have as many *p-values* as iterations *i* and repetitions of subsampling $f_i$ that are of the form:

3 $$\begin{aligned} \mathscr {P}_{i} = \left\{ (n_{i} ,p_{i}^{j}) , j \in [1,...,f_{i}] \right\} , n_{i} \in \mathscr {N}, f_{i} \in \mathscr {F}, \end{aligned}$$

where $\mathscr {N}$ is a grid of natural numbers and $\mathscr {F}$ is also a grid of the same nature which encompasses the number of *p-values* $f_i$ computed for sample size $n_i$. Thus, the computational cost of MCCV is reduced without losing information. Further details are given in the description of the MCCV routine in the Supplementary Information. Note that this procedure is similar to the upstrap^18^ using an increasing fraction of the sample.

The procedure is applied using random populations from different normal distributions. We distinguish two different situations: either the obtained *p-value* distribution is uniform, so the mean *p-value* of $\mathscr {P}_{i}$ is constant for any *i* (Supplementary Fig. S2a,b in the Supplementary Information); or the mean *p-value* tends to decrease when the sample size *n* increases (Supplementary Fig. S2c–f in the Supplementary Information). In other words, *p*(*n*) can be expressed as a continuous function by assuming that *n* is a positive real number. In order to illustrate that *p*(*n*) can be approximated by an exponential function as stated in Eq. (2), we proceed in two different ways. First, locally weighted scatter plot smoothing (LOWESS)^19^ is used to depict the exponential decay of the curve when *n* tends to infinity. Second, we verify that the ratio between the first derivative of the so-obtained curve and the curve itself is constant, i.e.

4 $$\begin{aligned} \dfrac{p'(n)}{p(n)} = c \Longleftrightarrow p(n) = a\cdot e^{cn} \quad \text {where } a, c \in \mathbb {R}. \end{aligned}$$

The LOWESS approximation is calculated with the mean *p-values* for each sample size *n*: for each iteration *i*, each set of $\mathscr {P}_{i}$ values is averaged to obtain the empirical estimation of the function *p*(*n*) at $n=n_i$ (red markers in Supplementary Fig. S2 in the Supplementary Information). Then, a smooth curve is fitted to these values using LOWESS, which shows that *p*(*n*) in Eq. (2) is appropriate due to its exponential shape (Fig. 2b,c). Collecting the values *p*(*n*) of the LOWESS fit, the ratio $\dfrac{p'(n)}{p(n)}$ is calculated (Fig. 2d,e). Most of the ratios verify the condition in Eq. (4). In Fig. 2e, we show cases in which it is more challenging to decide whether there exists a statistical difference, as for instance, when $\mathscr {N}(0,1)$ and $\mathscr {N}(0.1,1)$ are compared. When *p*(*n*) is very small, the ratio $\dfrac{p'(n)}{p(n)}$ has more outliers, especially when the sample size *n* is small. This can be observed when comparing $\mathscr {N}(0,1)$ with $\mathscr {N}(0.75,1)$, $\mathscr {N}(1,1)$, $\mathscr {N}(2,1)$ and $\mathscr {N}(3,1)$ (Fig. 2e). These are extreme cases in which there exist clear differences between populations and therefore, *p-values* are close to zero most of the time.

As we have proved above that *p*(*n*) in Eq. (2) is a good choice, an exponential curve is fitted to all the pairs of values $\mathscr {P}_{i}$ calculated with MCCV (Fig. 2b,c). Both LOWESS and exponential curves are very close to each other, even if the former was fitted using the mean values of each group $\mathscr {P}_{i}$ and the latter using the entire $\mathscr {P}_{i}$ set. An exponential fit is more suitable in this case as it is calculated with all the values obtained through MCCV and only outputs positive values by definition. A LOWESS approximation can occasionally lead to biased negative values, such as when $\mathscr {N}(0,1)$ and $\mathscr {N}(0.75,1)$ are compared while the *p-values* are positively defined. Note that as $p(n) \rightarrow 0$ when $n \rightarrow \infty$, $c<0$ necessarily in Eq. (4). Therefore, we assume from now on that *p-values* for different sample sizes can be expressed as an exponential function of the form in Eq. (2). The parameters *a* and *c* control the amplitude and the decay of the function *p*(*n*), respectively. If $c=0$, then the value of *p*(*n*) would be uniform in *a*: $p(n) = a$. As *p-values* are computed probabilities and the global maximum of *p*(*n*) is *a*, *a* belongs to the [0, 1] interval.

### Distance to the $\alpha$-level of statistical significance

**Figure 3:**
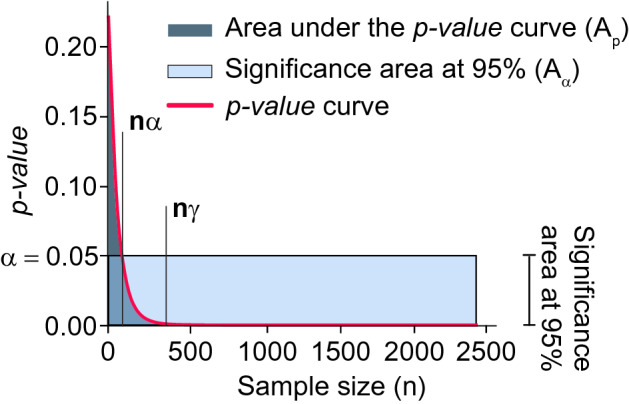
Comparison of a $95\%$ of confidence level ($\alpha = 0.05$) and an *n*-dependent *p-value* curve. The parameter $n_\alpha$ represents the minimum sample size to detect statistically significant differences among compared groups. The parameter $n_{\gamma }$ represents the convergence point of the *p-value* curve. When the *p-value* curve expresses practical differences, the area under the red curve ($A_{p(n)}$) is smaller than the area under the constant function $\alpha = 0.05$ ($A_{\alpha = 0.05}$) when it is evaluated between 0 and $n_{\gamma }$.

**Figure 4:**
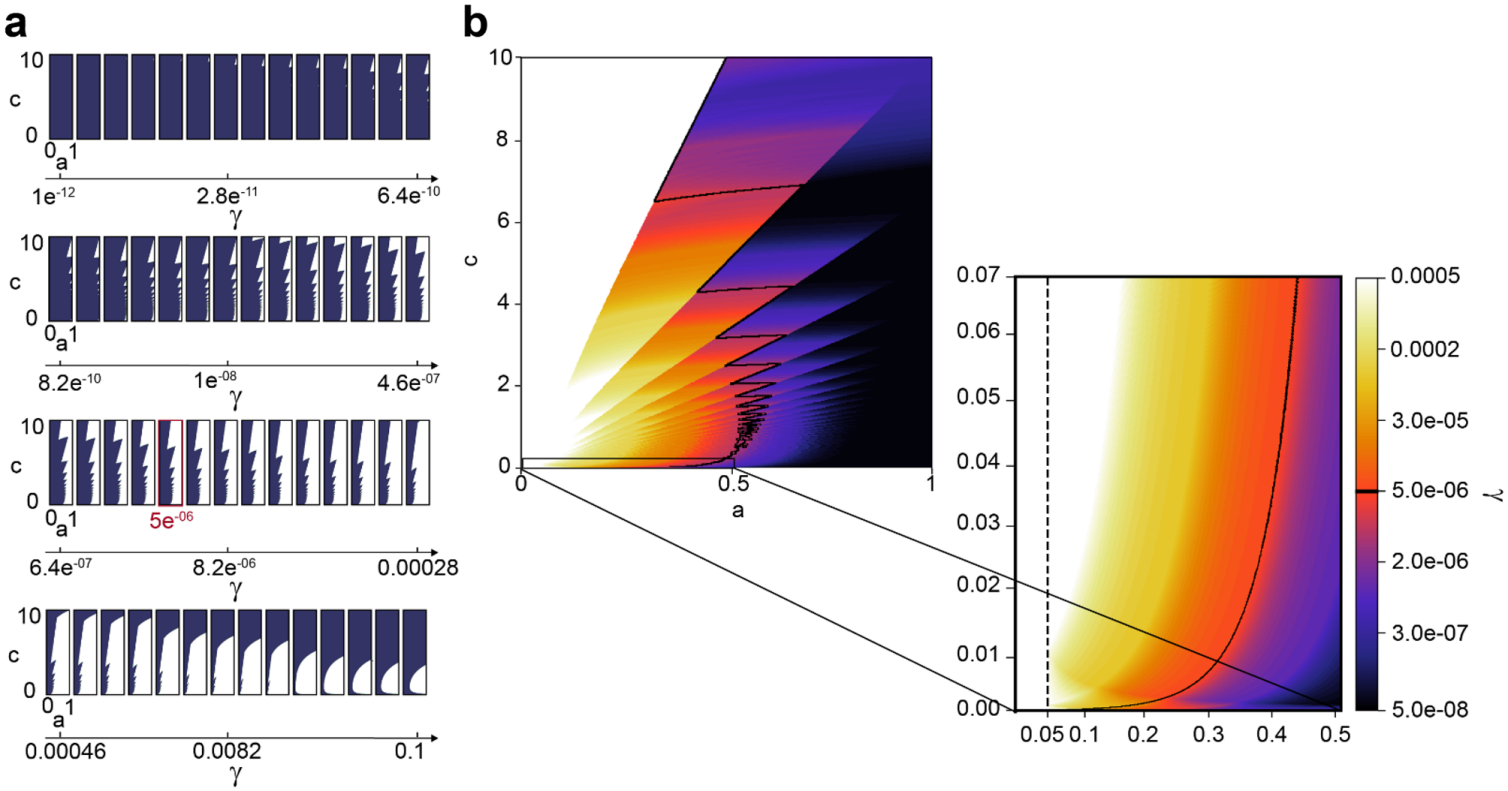
Decision index $\theta _{\alpha =0.05 , \gamma }$ for different values of parameters *a* and *c* in the function $ae^{-cn}$ and threshold $\gamma$: (**a**) Each of the subplots is drawn for a specific value of $\gamma$, being the dark area the cases for which we conclude that there are meaningful differences ($\theta _{\alpha =0.05 , \gamma }=1$), and white area the rest of the cases $\theta _{\alpha =0.05 , \gamma }=0$; (**b**) Colors in the image correspond to the values of $\gamma$ for which $\delta _{\alpha =0.05 , \gamma }=0$. The black frontier shows $\delta _{\alpha =0.05 , \gamma =5 e^{-06}}=0$ (red box in (**a**)). All the values of *a* and *c* for which $\theta _{\alpha =0.05 , \gamma =5 e^{-06}}=1$ (practical differences) lie on the left side of this limit and, the rest, on the right. The plots shown in (**a**) show the influence of the parameter $\gamma$ in a wide range of values, while the plots shown in (**b**) are limited to the range of values we find in this posterior experiment. The vertical dashed line indicates the cases $a=0.05$ which are the cases in which *p*(*n*) outputs a $95\%$ statistically significant value.

Because hypothesis tests are based on probabilities, there is always a chance of making a wrong decision. The ideal case would be that such a decision does not depend on the size of the sample gathered to carry out the test. In other words, $\alpha \cdot 100\%$ of *p-values* computed from different samples are lower than $\alpha$ when the null hypothesis is true (Type I error). Hence, we claim that whenever there exist clinically meaningful differences between two samples, *p*(*n*) reaches $\alpha$ rapidly. So, the values of *p*(*n*) are mostly distributed in a range smaller than $\alpha$. Therefore, we compare all the values of the curve *p*(*n*) with $\alpha (n) = \alpha$. In the discrete case, we would evaluate $\alpha - p(n_i)$ for each index *i* and sum all the results: if the sum is positive, then *p*(*n*) is smaller than $\alpha$ most of the time. In the continuous case, this sum is obtained by integrating the difference

5 $$\begin{aligned} \delta _{\alpha } \left( n \right) = \int \left( \alpha -p(n)\right) dn = A_{\alpha (n)} - A_{p(n)}, \end{aligned}$$

where $A_{\alpha (n)}$ is the area under the constant function $\alpha$ and $A_{p(n)}$ is the area under the estimated *p-values*’ curve, (Fig. 3). A positive $\delta _{\alpha }(n)$ implies that $A_{\alpha (n)}$ is larger than $A_{p(n)}$, i.e. most of the values in *p*(*n*) are below the significance threshold; a negative $\delta _{\alpha }(n)$ implies the opposite.

As shown in the next paragraphs, Eq. (5) aims to quantify and evaluate the distribution of *p-values* (i.e., the distribution of $\{(n, p(n)), n \in \mathbb {R}^+\}$ taking into account two aspects, whether (1) most of the *p-values* are smaller than $\alpha$ and (2) the decay of *p*(*n*) is fast.

### Mathematical formulation of the decision index

By means of the estimated parameters *a* and *c* in the expression of *p*(*n*) in Eq. (2), the measure $\delta _{\alpha } \left( n \right)$ (Eq. 5) can be rewritten as follows

6 $$\begin{aligned} \delta _{\alpha }(n) =\alpha \cdot n - \dfrac{a}{c}\left( 1-\mathrm {e}^{-cn}\right) . \end{aligned}$$

Due to the bounds *a* and *c*, $\delta _{\alpha }(n)$ is well-defined. However, whenever *n* tends to infinity, $\delta _{\alpha }(n)$ will always be positive and it tends to infinity.

7 $$\begin{aligned} \lim \limits _{n\rightarrow \infty } \delta _{\alpha }(n) \approx \lim \limits _{n\rightarrow \infty } \left(\alpha n- \dfrac{a}{c}\right)\rightarrow \infty . \end{aligned}$$

Also, from a practical perspective, the area of interest to evaluate the decay of *p*(*n*) is that enclosed between zero and its convergence point *n*: $\left| p'(n) \right| \approx 0$. Namely, a relevant sub-sample of size *n* can be computed as

8 $$\begin{aligned} n_{\gamma } = arg\min _{n} \left\{ \left| p'(n) \right| < \gamma \right\} , \end{aligned}$$

where $\gamma$ is the threshold chosen to determine the convergence point (Fig. 3). Finally, $\delta _{\alpha , \gamma }$ is now formally defined as

9 $$\begin{aligned} \delta _{\alpha , \gamma } = A_{\alpha (n_{\gamma })} - A_{p(n_{\gamma })} = \alpha \cdot n_{\gamma } - \dfrac{a}{c}\left( 1 - \mathrm {e}^{-cn_{\gamma }}\right) . \end{aligned}$$

The computation of $\delta _{\alpha , \gamma }$ enables the identification of a rapid convergence to zero at small *n* induced by the high slope of *p*(*n*), which is indicative of the existence of interesting differences among the groups being tested.

The decision index we propose, $\theta _{\alpha ,\gamma }$, is defined as

10 $$\begin{aligned} \theta _{\alpha ,\gamma } = {\left\{ \begin{array}{ll} 1, &{} \delta _{\alpha ,\gamma }\ge 0 \\ 0, &{} \text{ otherwise } \end{array}\right. }, \end{aligned}$$

where $\delta _{\alpha ,\gamma }$ follows Eq. (9), where $\theta _{\alpha ,\gamma } = 1$ means that there are practical differences among the compared samples.

#### Delimiting the convergence of the curve *p*(*n*)

The proposed approach depends on two thresholds: (1) significance threshold $\alpha$ and (2) the convergence threshold $\gamma$. The former measures the level of statistical significance, while the latter controls decisions. Therefore, the only critical threshold to discuss in this work is $\gamma$.

The rules to follow for the selection of the threshold $\gamma$ are:

- The parameter *a* is the maximum value that *p*(*n*) can take. Also, *p*(*n*) may not achieve *a* since *n* should be strictly greater than 0. Therefore, if *a* is smaller than $\alpha$, then $\theta _{\alpha ,\gamma } = 1$ for any $\gamma$ given.
- As $\delta _{\alpha }(n)$ tends to infinity with *n*, the smaller the value of $\gamma$ is set, the larger $n_{\gamma }$ will be and $\theta _{\alpha ,\gamma } = 1$ becomes more likely.
- The values of $\gamma$ should be small: $\alpha$ is considered the maximum probability allowed of making a Type I error (rejecting the null hypothesis when it is true) and *p*(*n*) values are constantly compared with it. It seems reasonable to compare the slope of *p*(*n*) at the convergence point with a value smaller than $\alpha$, which is usually smaller than 0.1.

Eq. (8) implies

11 $$\begin{aligned} \left| p' (n)\right| = \left| -cp(n)\right|< \gamma \Longleftrightarrow p(n) < \dfrac{\gamma }{\left| c\right| }. \end{aligned}$$

So, if $\gamma$ is chosen such that $\dfrac{\gamma }{\left| c\right| }$ is greater than $\alpha$, the assumption that *p*(*n*) has arrived to a convergence point equivalent to zero would vanish. Therefore, our claim is that $\dfrac{\gamma }{\left| c\right| }<\alpha$ with at least, $\gamma \le 0.1$.

### Data characterization in stable and uncertain cases

The threshold $\gamma$ controls severe decisions and it is limited to $\dfrac{\gamma }{\left| c\right| } < \alpha$ with at least $\gamma \le 0.1$. In this section we study the range $\gamma \in [ 1e^{-12}, 0.1]$ to see its effect on the decision index $\theta _{\alpha ,\gamma }$. Namely, the lower $\gamma$ is set, the larger the value *n* is to determine *p*(*n*)’s convergence. Hence, when $\gamma$ is small, then the decision index will determine that practical differences among groups are more likely, becoming then less strict. In Fig. 4a, we show the dynamics of $\theta _{0.05,\gamma }$ when $\gamma$ changes: the dark area ($\theta _{0.05,\gamma }=1$) increases inversely to $\gamma$ until a certain value from which the method becomes more restrictive with the rejection of the null hypothesis ($\gamma \ge 0.0005$), showing that the chances for which the null hypothesis is rejected increase as well. Moreover, the limit between dark and light ($\theta _{0.05,\gamma }=0$) areas is precisely the curve $\delta _{\alpha , \gamma }= 0$. The value of $\gamma$ determines this curve and therefore, the conditions for which $\theta _{0.05,\gamma } = 1$ (dark area) and $\theta _{0.05,\gamma } = 0$ (light area). In Fig. 4b, we illustrate the condition $\delta _{\alpha , \gamma }=0$ when $\alpha =0.05$, as a function of *a*, *c* and $\gamma$. The case $\gamma = 5e^{-06}$ is underlined in black.

**Figure 5:**
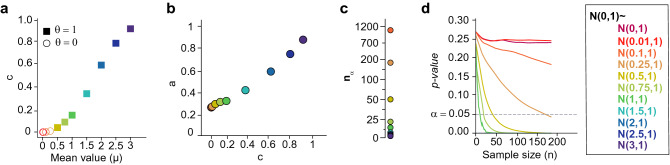
Estimation of the *p-value* as a function of the size (*p*(*n*)) enables the correct discrimination between conditions. (**a**) The decay of *p*(*n*) (parameters *a* and *c* of the exponential fit) increases with the mean value of the normal distribution being compared with $\mathscr {N}(0,1)$. The larger the distances between the means of the distributions, the higher the decay of the exponential function (Table 1). (**b**) The empirical estimation of *p*(*n*) with small datasets enables the detection of the most extreme cases: those in which the null hypothesis can be accepted, and those in which it clearly cannot. (**c**) The minimum data size needed to obtain statistical significance ($n_{\alpha }$) is inverse to the mean value of the normal distributions being compared. (**d**) The faster the decay of *p*(*n*), the stronger the statistical significance of the tested null hypothesis. For $\gamma = 5e^{-06}$, $\theta _{\alpha =0.05, \gamma =5e^{-06}}=1$ whenever the mean value of the normal distribution compared with $\mathscr {N}(0,1)$ is larger than 0.5 (Table 1).

There exist some points (*a*, *c*) for which $\theta _{0.05,\gamma } = 0$ is independent of $\gamma$. A clear example is the case in which $a\ge \alpha$ and $c\approx 0$. These cases represent the situation in which the null hypothesis in the test cannot be rejected with a statistical significance of level $\alpha$. For instance, when $\mathscr {N}(0,1)$ is compared with $\mathscr {N}(0,1)$ or $\mathscr {N}(0.01,1)$, Fig.5 or Supplementary Fig. S2a,b in the Supplementary Information. Likewise, if $a\le \alpha$ or *c* is large enough, the null hypothesis is always rejected with a statistical significance of level $\alpha$. For instance, when $\mathscr {N}(0,1)$ is compared with $\mathscr {N}(2,1)$ or $\mathscr {N}(3,1)$, Fig. 5 and Supplementary Fig. S2e,f in the Supplementary Information. Therefore, the proposed methodology is useful for the cases in which there is high uncertainty about the rejection of the null hypothesis.

The proposed methodology allows us to classify the decisions on the differences among the groups of observations by their level of uncertainty. Namely, if the differences can be considered relevant from a practical perspective or not. The parameters of the exponential curve in Eq. (2) determine the axis of any of the plots in Fig. 4. Therefore, once an exponential curve is fitted and parameters *a* and *c* are estimated, it is possible to know in which position of the graph the case of study is: clear cases will always be close to the left (there are not differences) or to the right (there are practical differences) side of the graphs in Fig. 4, while most unstable or unclear cases will be placed in the middle. Therefore, with this method, it is possible to determine if there are clinically significant differences or not. When these differences are not sufficiently clear, it might be necessary to perform a deeper study.

An intuitive interpretation of statistically significant differences between two groups (the classical threshold *p-value*$<\alpha$) is that the CI of the means of the groups do not overlap. The width of the CI decreases when the size of the data increases^20^, Fig. 1. The rest of the section is devoted to study how large two populations must be in order to obtain non-overlapping CI. Interestingly, the estimation of the function *p*(*n*) allows us to determine the specific minimum value of *n*, $n_{\alpha }$, for which *p*(*n*) is lower than the significance level $\alpha$ (Fig. 4). This value is the solution to the equation

12 $$\begin{aligned} \alpha = ae^{cn_{\alpha }}. \end{aligned}$$

As computed, $n_{\alpha }$ represents the minimum sample size needed to obtain a statistically significant *p-value*, in case it exists. In other words, reproducing an experiment with $n_{\alpha }$ samples assures the rejection of the null hypothesis. The estimated $n_{\alpha }$ allows to assess the evidence against the null hypothesis. If $n_{\alpha }$ is small, the strength of the statistical difference is very clear and two populations are distinguishable. The parameters *a* and *c* in Eq. (12) are obtained empirically through MCCV so they can introduce some bias in the calculation of $n_{\alpha }$. Hence, a better estimator of $n_{\alpha }$, $\hat{n}_{\alpha }$ , can be computed using the *p-values* obtained directly from the data and their variance

13 $$\begin{aligned} \hat{n}_{\alpha } = arg\min _{n_{i}} \left\{ ( \bar{p_{i}}- \sigma _{\bar{p_{i}}})<\alpha \right\} , \end{aligned}$$

where $\bar{p_{i}}$ represents the mean of the set of values $\mathscr {P}_{i}$ (MCCV) and $\sigma _{\bar{p_{i}}}$, the mean standard error (SEM), which is included to correct for the variability of the estimated *p-values*. The estimator $\hat{n}_{\alpha }$ is limited to those cases in which the data is large enough: if the size of the data is smaller than $n_{\alpha }$, then $\hat{n}_{\alpha }$ cannot be computed (Fig. 5b). As $\hat{n}_{\alpha }$ is more restrictive than $n_{\alpha }$, its value will always be slightly larger (Table 1). The values in Table 1 are similar to those values in Fig. 5c for which the CIs do not overlap.

### Test of reliability

Unlike many computational methods, the analysis of statistical significance of the differences between two groups cannot be evaluated by means of Ground Truth data, simulations or human-made annotations. Nonetheless, it is possible to determine the robustness on the reproducibility of the results. Namely, whether the decision taken about the stated null-hypothesis ($\theta _{\alpha , \gamma }$) is maintained when the experiment is repeated. To do so, we test our method using simulated normal distributions.

Any data diagnosis carried out with the proposed method depends on the value $\gamma$ chosen and the limitations posed by its computational intensive nature. As done at the beginning of this work, we compare the normal distribution $\mathscr {N}(0,1)$ with $\mathscr {N}(0.01,1)$, $\mathscr {N}(0.1,1)$, $\mathscr {N}(0.25,1)$, $\mathscr {N}(0.75,1)$, $\mathscr {N}(1,1)$, $\mathscr {N}(2,1)$ and $\mathscr {N}(3,1)$. We should obtain $\theta _{\alpha ,\gamma }=1$ when comparing the most similar distributions such as $\mathscr {N}(0,1)$ and $\mathscr {N}(0.01,1)$. In contrast, we should get $\theta _{\alpha , \gamma }=0$ when comparing the most different distributions, such as $\mathscr {N}(0,1)$ and $\mathscr {N}(2,1)$.

To evaluate the effect of $\gamma$, *p*(*n*) is simulated for all pairs of normal distributions and it is compared with a significance level of $\alpha =0.05$ using different values of $\gamma$ (Supplementary Table S7 in the Supplementary Information). The lower the convergence criterion $\gamma$ is, the less restrictive the diagnosis is (Fig. 4). Using the simulated data, the range of $\theta _{0.05,\gamma }$ values obtained let us recommend a value for $\gamma$. When $\mathscr {N}(0,1)$ and $\mathscr {N}(0.1,1)$ are compared with a small $\gamma$ ($\gamma = 2.5\times 10^{-6}$), the decision index $\theta _{\alpha =0.05, \gamma = 2.5\times 10^{-6}}$ indicates that there exist meaningful differences among both distributions, which is the opposite of what we expected. If the value of parameter $\gamma$ increases, the decision index will output that the two compared groups do not display meaningful differences in those cases in which there is a larger uncertainty about this decision. For instance, when $\mathscr {N}(0,1)$ and $\mathscr {N}(0.25,1)$ are compared with $\gamma = 5 \times 10^{-5}$, $\theta _{\alpha =0.05, \gamma = 5\cdot 10^{-5}} = 0$ (See Supplementary Table S7). However, the latter is not straightforward for two reasons: $\delta _{\alpha =0.05,\gamma =5 \times 10^{-5}} = -5.84$ (small difference) and $\hat{n}_{\alpha } = 186$ (few samples to observe statistically significant differences). As it is shown in Fig. 4, a value $\gamma >5e^{-04}$ results in $\theta _{\alpha =0.05, \gamma }=0$ for all the cases in Table 1. Note that the values of the function *p*(*n*) are enclosed in the [0, 1] range and that $\gamma$ is used to determine where the *elbow* of this function *p*(*n*) lays, i.e. the convergence point. Therefore it is reasonable to use the same value of $\gamma$ regardless the data that is being analyzed. With the results of the evaluation, we strongly recommend the use of $\gamma =5e^{-06}$. Indeed the decisions about having interesting differences among the compared groups are robust to the changes in the value of $\gamma$ in both simulated and experimental data (Supplementary Tables S7 and S9 in the Supplementary Information). Moreover, uncertain decisions can be easily spotted by small $\delta _{\alpha , \gamma }$ and $\hat{n}_{\alpha }$ values.

**Table 1:** Results of comparing the normal distribution $\mathscr {N}(0,1)$ with other simulated normal distributions. Parameters of the function *p*(*n*) after the exponential fit with $\alpha =0.05$ and $\gamma =5e^{-06}$, for the comparison of a normal distribution with mean value 0 and standard deviation 1, and normal distributions of mean values 0, 0.01, 0.1, 0.25, 0.5, 0.75, 1, 1.5, 2, 2.5 and 3.

Comparison	*a*	*c*	$\hat{n}_{\alpha }$	$n_{\alpha }$	$\theta _{0.05, 5\cdot 10^{-6}}$
$\mathscr {N}(0,1) \sim \mathscr {N}(0,1)$	0.256	0.000	$\infty$	39, 599	0
$\mathscr {N}(0,1) \sim \mathscr {N}(0.01,1)$	0.255	0.000	$\infty$	44, 237	0
$\mathscr {N}(0,1) \sim \mathscr {N}(0.1,1)$	0.257	0.000	1192	988	0
$\mathscr {N}(0,1) \sim \mathscr {N}(0.25,1)$	0.263	0.010	185	165	1
$\mathscr {N}(0,1) \sim \mathscr {N}(0.5,1)$	0.286	0.042	47	41	1
$\mathscr {N}(0,1) \sim \mathscr {N}(0.75,1)$	0.304	0.091	20	19	1
$\mathscr {N}(0,1) \sim \mathscr {N}(1,1)$	0.313	0.152	13	12	1
$\mathscr {N}(0,1) \sim \mathscr {N}(1.5,1)$	0.411	0.344	7	6	1
$\mathscr {N}(0,1) \sim \mathscr {N}(2,1)$	0.579	0.599	5	4	1
$\mathscr {N}(0,1) \sim \mathscr {N}(2.5,1)$	0.738	0.794	4	3	1
$\mathscr {N}(0,1) \sim \mathscr {N}(3,1)$	0.867	0.924	4	3	1

To test the generality of these results, the same procedure was repeated several times by changing the samples of the normal distributions being compared. Hence, it is possible to provide a probability of how often the resulting $\theta _{\alpha , \gamma }$ would be the same as the one stated in Table 1. Additionally, the presented method has its limitations in the computational time needed to perform MCCV iterations: the more iterations we compute the longer the process will take. Moreover, the accuracy of any estimated *p*(*n*) depends on the maximum sample size $n=n_i$ considered and *p-values*, $\mathscr {P}_i$, that the program can evaluate. Therefore, we also tested the results of the method when the number of iterations in MCCV is reduced. Overall, a rate between obtaining exactly the same result or a different one under any change of the previous conditions was calculated (Supplementary Table S8 in the Supplementary Information). The rate is given as a percentage value. The closer the percentage gets to 100, the more robust and general the result will be. We can confirm that the results are most of the time the same as the ones given in Table 1 when $\gamma =5e^{-06}$. The only critical case is the comparison $\mathscr {N}(0,1)\sim \mathscr {N}(0.5,1)$ when few $n_i$ points in $\mathscr {N}$ are used to estimate *p*(*n*). The last procedure was repeated using the real data from Experiment 1 (study of the effect of Taxol in the cell body and protrusions morphology) (Supplementary Tables S9 and S10 in the Supplementary Information). Even with more complex and noisier data, the results obtained show that the method is stable and robust. All technical details about these computations are given in the Supplementary Information.

### Ethics declarations

There is no direct human participation in the study.

## Materials

We describe the non-published dataset that corresponds to the first real application example in the reported results. Phase contrast microscopy images of a human invasive ductal carcinoma (MDA-MB-231) cell line were acquired. The set-up used was composed by a Cascade 1K CCD camera (Roper Scientific), mounted on a Nikon TE2000 microscope with a 10$\times$ objective lens. Cells were embedded in 3D collagen type I matrix at 100.000 cells/mL. The time lapse videos were recorded every two minutes with a focus plane of at least 500 away from the bottom of the culture plates to diminish edge effects^21^. Three different groups of cells were analyzed: control and treated with fresh media at 1 nM Taxol and 50 nM Taxol. Ten videos of 16.5 h (500 frames of $809 \times 810$ with a resolution of 0.806) each were analyzed per group. All videos were automatically processed using a convolutional neural network (U-net^22^) to get binary masks for the cell bodies and their protrusions. The resulting semantic segmentation corresponds uniquely to focused cells in the image. For each of these cells, their body and protrusions are segmented. See some examples of the resulting segmentation in Supplementary Fig. S4 in the Supplementary Information. Using the segmentations, eight different morphological measurements were calculated: cell body size (CS), cell body perimeter (CP), cell body roundness (CR), cell with at least one protrusion (Pb), protrusion size (PS), protrusion perimeter (PP), protrusion length (PL) and protrusion diameter (PD) (Supplementary Table S1 in the Supplementary Information). Further information about the distribution of each of the measurements is given in the Supplementary Information.

## Results

The decision index, descriptive parameters *a* and *c* (Eq. 2) and minimum data size $n_{\alpha }$ provide an intuition about the distance between the distributions of the datasets being compared. To illustrate this, sample data generated from different normal distributions were compared using the Mann–Whitney U statistical test^15^, assuming a significance level $\alpha$ of 0.05 (Table 1). When $\mathscr {N}(0,1)$ is compared with $\mathscr {N}(0,1)$, $\mathscr {N}(0.01,1)$ and $\mathscr {N}(0.1,1)$, $\theta _{\alpha =0.05, \gamma =5e^{-06}}$ is null; so those distributions are assumed to be equal if our approach is used. In the remaining comparisons though, according to our approach, $\theta _{\alpha =0.05, \gamma =5e^{-06}}=1$, thus there exist differences between $\mathscr {N}(0,1)$ and $\mathscr {N}(\mu ,1)$ for $\mu \in \left[ 0.25, 3\right]$ (Fig. 5a). Together *a* and *c* provide a spatial representation of the distance between all the normal distributions and $\mathscr {N}(0,1)$ (Fig. 5a,b). Likewise, when $\mathscr {N}(0,1)$ is compared with $\mathscr {N}(\mu ,1)$ for $\mu \in \left[ 0.1, 3\right]$ , the value of $n_{\alpha }$ increases as the mean value $\mu$ decreases. Indeed, $n_{\alpha }$ cannot be determined when $\mathscr {N}(0,1)$ is compared with $\mathscr {N}(0,1)$ and $\mathscr {N}(0.01,1)$, as the null hypothesis in this case is true. Therefore, *p*(*n*) is a constant function, which represents the uniform distribution of *p-values* under the null hypothesis (Fig. 2b,c).

**Figure 6:**
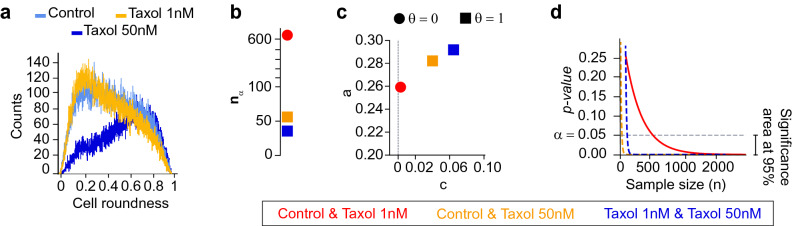
Breast cancer cells (MDA-MB-231) were cultured in collagen and imaged under a microscope to determine if cells change shape when a chemotherapy drug (Taxol) is administered. Three different groups were compared: control (non-treated) cells, cells at 1 nM and at 50 nM Taxol. (**a**) The cell roundness distribution of control cells (non-treated) and cells treated at 1 nM Taxol have lower values than that of cells treated at 50 nM. (**b**–**d**) The three groups were compared, the *p-values* were estimated and *p*(*n*) was fitted for each pair of compared groups. When Taxol at 50 nM is evaluated (blue and yellow dashed curves), $n_{\alpha }$ is lower and the decay of *p*(*n*) is higher (*a* and *c* parameters in Eq. 2), i.e. it decreases much faster than the one corresponding comparison of control and Taxol at 1 nM (orange curve) indicating the presence of meaningful differences between cells treated at 50 nM Taxol and the remaining groups.

**Figure 7:**
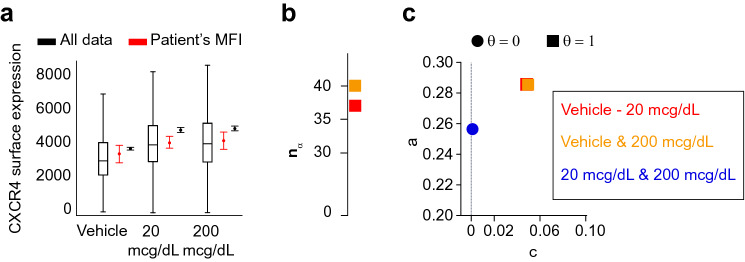
Flow cytometry data recorded to determine the transcriptional changes induced by the in vivo exposure of human eosinophils to glucocorticoids. (**a**) The entire dataset has a wider range of values (black box-plots) and a smaller $95\%$ confidence interval around the mean (black error-plots) than the distribution obtained when the median fluorescence intensity (MFI) is calculated by each of the 6 subjects (red error-plots). (**b**,**c**) There is an increase of the surface expression of CXCR4 when human eosinophils are exposed to 20 or 200 mcg/dL of Methylprednisolone. Namely, (**b**) the minimum size $n_{\alpha }$ is low and the decision index $\theta _{\alpha =0.05, \gamma =5e^{-06}}=1$ when any of those conditions are compared with the vehicle condition. The minimum size $n_{\alpha }$ when eosinophils are treated (blue circle) is not shown as it has infinite value. (**c**) The decay parameters *a* and *c* are almost the same in those two cases, so the markers co-localize (Supplementary Information).

**Figure 8:**
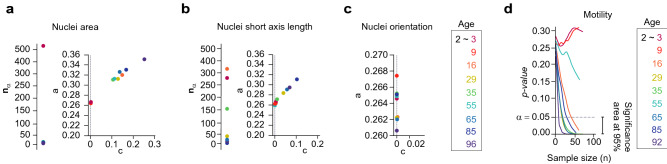
(**a**,**b**) The morphology of 2-year-old human cells is compared with the morphology of 3, 9, 16, 29, 35, 45, 55, 65, 85 and 96-year-old human cells. For both, (**a**) nuclei area and (**b**) nuclei short axis measures, the minimum size $n_{\alpha }$ and the decay *a* change proportionally with the age of the donor. (**c**) The nuclei orientation does not characterize the age of the human donors for all the comparisons; the parameter *c* is null, and therefore, *p*(*n*) is constant. (**d**) The analysis of a small dataset is enough to determine that the total diffusivity can characterize the cellular aging in humans. The total diffusivity of 2, 3 and 9-year-old human cells are equivalent, while it differs when compared to cells from older human donors.

To prove the benefit of the proposed method, we tested its different functionalities on published and non-published real data from biological experiments. The first real application of the method consists of discriminating between experimental conditions. In this case, we wanted to determine whether cancer cells cultured in 3D collagen matrices and imaged under a light microscope changed shape after administration of a chemotherapeutic drug (Taxol) (details about data collection and processing are given in the Supplementary Information). This information is relevant as it could give an indication about the metastatic potential of the treated cells^23,24^. Three different groups were compared: control cells ($\approx 78,000$ cells), and cells treated with 1 nM ($\approx 75,000$ cells) and 50 nM Taxol ($\approx 46,000$ cells) respectively. The three groups were compared pairwise and none of the groups is normally distributed. Here we show the case for the *Roundness* variable, although the analysis for the remaining ones is given in the Supplementary Information. Cells exposed to low concentrations of Taxol (1 nM) remained elongated (low roundness index, which suggests higher metastatic behavior of the cells), i.e. $\theta _{\alpha =0.05, \gamma =5e^{-06}} = 0$ for the comparison between control cells and those treated with Taxol at 1 nM. However, when the dose was increased to 50 nM Taxol, cells became circular (lower metastatic behavior); therefore $\theta _{\alpha =0.05, \gamma =5e^{-06}}=1$ when comparing cells treated with 50 nM Taxol versus control cells, or cells treated with 1 nM Taxol (Fig. 6 and Supplementary Table S3 in the Supplementary Information).

Secondly, we analyzed the flow cytometry data used by Ref.^25^ to determine the transcriptional changes induced by the in vivo exposure of human eosinophils to glucocorticoids. The eosinophils belong to 6 different healthy human subjects. The proposed method allowed us to discriminate between treated and untreated eosinophils using the entire dataset. For that, we analyzed the eosinophil surface expression of the gene CXCR4 2 h after the exposure to 20 and 200 mcg/dL of Methylprednisolone. With the estimation of the function *p*(*n*) (Eq. 2), it is possible to conclude that the exposure of eosinophils to glucocorticoids causes a differential expression of CXCR4 (Fig. 7), i.e. $\theta _{\alpha =0.05, \gamma =5e^{-06}}=1$ for the comparison between vehicle and eosinophils treated with 20 and 200 mcg/dL (Supplementary Table S6 in the Supplementary Information). Indeed the conclusion is the same as the one made in Ref.^25^, where only the median fluorescence intensity of the data from each subject was calculated and the resulting 6 data points were compared (Fig. 7). However, the latter approach can lead to false conclusions when the data distributions are different for each group or when their standard deviations are large.

The last use of the method we propose here consists of analyzing whether a single specific feature of the data (variable) can fully characterize the problem at hand. Many different biomolecular and biophysical features of human cells were analyzed in Ref.^26^ with the aim of predicting cellular age in healthy humans. For the experiments in that study, the cells were collected from human subjects from 2 to 96-years-old. The method proposed in this manuscript can help to decide which features contain relevant information about subject aging. To show that, we re-analyzed the information of nuclei morphology and cell motility collected by Ref.^26^. The former is a large dataset and the latter is a small one. The information of 2-year-old human cells (the youngest one) was compared with the rest of the ages. The decay of *p*(*n*) in cell nuclei area and short axis length show that these nuclei morphology parameters are directly related to the age of human cells. The parameter *c* (Eq. 2) of the orientation of the cell nuclei is null in all cases, which indicates that this measure does not contain information about aging (Fig. 8c and Supplementary Table S5 in the Supplementary Information). It is relevant to observe that the pattern in the plots of *a* and *c* indicates whether the analyzed feature can characterize the age of the patients: increasing values of *a* and *c* as the age of the patients increases (cell nuclei area and short axis length, Fig. 8a,b) and *a* null *c* regardless the age of the patient (orientation of the cell nuclei, Fig. 8c). The estimated function *p*(*n*) for the total diffusivity of the cells of 2-year-old and 3-year-old human donors shows that even if a larger dataset was given, the result will remain the same (Fig. 8b and Supplementary Table S4 in the Supplementary Information). Namely, *p*(*n*) does not decrease, therefore, there is strong evidence that the null hypothesis is true (i.e. $\theta _{\alpha =0.05, \gamma =5e^{-06}}=0$, groups behave similarly). So, in this case, the analysis of a small number of cells is enough to conclude the non-rejection of the null hypothesis. The most extreme cases given by the differences between 2 and 96-year-old human donors, can also be detected without the need of large datasets, $n_{\alpha }=11$ (Fig. 8d). That is, the estimation of *p*(*n*) supports the decision about how many experimental samples need to be collected to conclude about the biological or clinical relevance of the differences between experimental groups.

The use of MCCV with large enough datasets guarantees robust estimators. In this case, different combinations of model parameters (MCCV iterations, used sample sizes and $\gamma$ value) where repeatedly tested to evaluate the variability of the decision index ($\theta _{\alpha , \gamma }$) and its sensitivity to the method set up (See “Test of reliability”). A larger $\gamma$ value results in a more restrictive decision index $\theta _{\alpha , \gamma }$ in the task of detecting interesting differences (Supplementary Tables S7 and S9 in the Supplementary Information). When the number of iterations of MCCV is drastically reduced, the decision index ($\theta _{\alpha , \gamma }$) shows instability only in those cases for which it is not clear that the groups differ from each other (Supplementary Table S8 and S10 in the Supplementary Information).

## Conclusion

The use of statistical hypothesis testing is largely extended and well established in the scientific research literature. Moreover, the number of statistically significant *p-values* reported in scientific publications has increased over the years^27^ and there exists a tendency among researchers to look for that combination of data that provides a *p-value* smaller than 0.05^9^. However, it has been shown here and also by Refs.^3–9^, that the assessment of the *p-value* has some drawbacks which can lead to spurious scientific conclusions. The data recorded from high-content, high-throughput studies, and the capacity of the computers to analyze thousands of numbers, has enabled us to enlighten the current uncertainty around the exploited *p-value*.

We report clear evidence about the well-known dependence of the *p-value* on the size of the data^3,12,28^. This particular feature of the *p-value* is used to characterize the differences among the groups of datasets being analyzed. Due to the lack of techniques that exploit the sensitivity of the *p-value* with respect to the sample size, we believe that our method will have a huge impact in the way scientists perform hypothesis testing.

With the proposed estimation of the decay of the *p-value* with the sample size, we provide a new perspective about hypothesis testing that prevents from treating the *p-value* as a dichotomous index. Using a simple mathematical formulation, an unbiased decision index $\theta _{\alpha , \gamma }$ is defined to enable good *praxis* in the same context as statistical hypothesis testing. The method takes advantage of large sample sizes to analyze the dependence of the *p-value* using cross validation. This approach provides stable measures that are robust to the noise in the data or the uncertainty around the decision making process. One of the advantages of our approach against computing effect sizes and CI, is mitigating the effect that the noise may have in the data. The analysis of the *p*(*n*) provides quantitative parameters to understand the relationship among the compared groups. As shown with the real experiments, visualizing the different exponential decay of *p*(*n*) supports a better understanding of biological information embedded in the data.

The presented method is applicable in any field of study beyond life-sciences as the classical NHST. Moreover, this methodology could be transferred to multiple comparison frameworks such as the ANOVA test by approximating the *p-value* function for each pair of comparisons. Although the analysis of simulated and real data was deployed using the Mann–Whitney U test, our methodology and programming library allow the inclusion of different tests such as the Student t-test or $\chi ^2$ test (see Supplementary Information and Supplementary Fig. S8). Likewise, our approach allows the analysis of heteroscedastic datasets as shown with the data from real experiments. The proposed approach used as a preliminary analysis, provides evidence about the existence (or not) of interesting differences from a practical perspective, even when large datasets are not available. Therefore, it supports the management of new data collection and can help researchers to reduce the cost of collecting experimental data.

The decision-index $\theta _{\alpha , \gamma }$ obtained with the proposed analytic pipeline relies on a new threshold called $\gamma$. Compared to the classical *p-value* and $\alpha$ threshold, the parameter $\gamma$ is mathematically constrained and $\theta _{\alpha , \gamma }$ is stable to its variations. Similarly, $n_{\alpha }$ is an effect size indicator, i.e. how different the samples are or how big this difference is (“Data characterization in stable and uncertain cases”). Additionally, the fitted parameters *a* and *c* that determine *p*(*n*) in Eq. (2), represent graphically how each of the conditions of an experiment relate to each other regarding the distribution of their values (Figs. 5a,b, 6, 7 and 8a–c). When the differences between the compared samples increase, the value of *a* and *c* increase as well regardless of the sample size, which suggests again, that *a* and *c* are indicators of the effect size.

The computational cost of the proposed data diagnosis increases proportionally with the number of groups to compare and the numerical setup of MCCV. Therefore, the optimization of the code and its connection to either a GPU or cloud computing is recommended. Overall, we advocate for the implementation of our pipeline in user-friendly interfaces connected to either cloud-computing or GPU. The code provided within this manuscript is built into the free software Python (https://github.com/BIIG-UC3M/pMoSS), so that anyone with limited programming skills can include any change to obtain a customized tool.

## Supplementary Information

Supplementary Information.

## Data Availability

The dataset belonging to the Experiment 1 and the code are available at https://github.com/BIIG-UC3M/pMoSS. The data used in Experiment 2 and 3 are provided within the corresponding publication as cited in the main text.
