# Supplementary material for: Use of the p-values as a size-dependent function to address practical differences when analyzing large datasets
Source: Sci Rep. 2021 Oct 22;11:20942. doi: 10.1038/s41598-021-00199-5 (PMC8536742; doi:10.1038/s41598-021-00199-5)
Supplement: Supplementary file 1 — Supplementary Information. [file 41598_2021_199_MOESM1_ESM.pdf]

Use of the *p-values* as a size-dependent function to address practical differences when analyzing large datasets

Supplementary Information

E. Gómez-de-Mariscal, V. Guerrero, A. Sneider, H. Jayatilaka, J. M. Phillip, D. Wirtz and A. Muñoz-Barrutia

Corresponding author: Arrate Muñoz-Barrutia
E-mail: mamunozb@ing.uc3m.es
Code availability at https://github.com/BIIG-UC3M/pMoSS

Contents

1 Evidence about the *p-value* and effect sizes being dependent of the sample size **1**

2 Technical details about the method **1**
A *p-values* tend to zero for large sample sizes . . . 1
B *p-values* as a function of the sample size . . . 4
C Estimation of *p-values* with Monte Carlo cross validation method . . . 4
D Assessment of minimum data size needed for statistical significance (n_α) . . . 5

3 Implementation details **6**

4 Experimental data **7**
A Experiment 1: Drug analysis on phase contrast microscopy data . . . 7
B Experiment 2: Drug analysis on flow cytometry data . . . 8
C Experiment 3: Cellular age characterization by means of biomolecular and biophysical properties . . . 9

5 Test of robustness **9**
A Test of robustness on theoretical data . . . 9
B Test of robustness on real data . . . 11
B.1 The *p-value* can be estimated by an exponential function . . . 11
B.2 Robustness of the convergence threshold and required computational load . . . 12

1. Evidence about the *p-value* and effect sizes being dependent of the sample size

For each sample size, we use Monte Carlo with simulated samples from normal distributions to calculate both the mean and its two sided 95% confidence interval (CI). Note that we distinguish between the theoretical CI S1 and the bootstrapped CI (Fig. 1 in the main manuscript). Following the same procedure as for the confidence intervals of the mean in the main manuscript (Section 2.1), two of the most common measures of effect sizes are also calculated: Cohen's d and Jensen-Shannon divergence and distance, Figure S1. We compute both, their mean value for all the computations at each fixed sample size and the bootstrapped two-sided 95% CI. Figure S1 does also display the maximum and minimum simulated values for each sample size. In these cases, there is an exponential convergence to the estimated value as well.

Likewise, Figure S2 provides graphical evidence that the *p-value* could be a function that depends on the sample size. In Fig. S2 different randomly generated normal distributions are compared using the Mann-Whitney U statistical test, (1) to illustrate that there exists a continuous inverse relation between *p-values* and n, i.e. *p-values* decrease when n increases, (2–4).

2. Technical details about the method

The main motivation of the study is that the *p-value* is misleading when working with large datasets as its value tends to zero. In Section A, we demonstrate for the particular cases of the Mann-Whitney U test, (1), and Student's t-test, (5), that indeed, the *p-value* tends to zero except for the cases in which the compared groups have the same distribution.

A. *p-values* tend to zero for large sample sizes. The statistic U of the Mann-Whitney U test, (1), is defined as $\min\{U_1, U_2\}$, where U_i follows the Eq. (1), being n_i the size of the dataset i and R_i its rank sum.

$$U_i = n_1 n_2 + \frac{n_i(n_i+1)}{2} - R_i, \quad i \in \{1, 2\}. \qquad [1]$$

When n_i are large enough, U follows a normal distribution, (1), with mean and standard deviation values, $\mu_{\mathcal{U}}$ and $\sigma_{\mathcal{U}}$ respectively, described by Eq. (2).

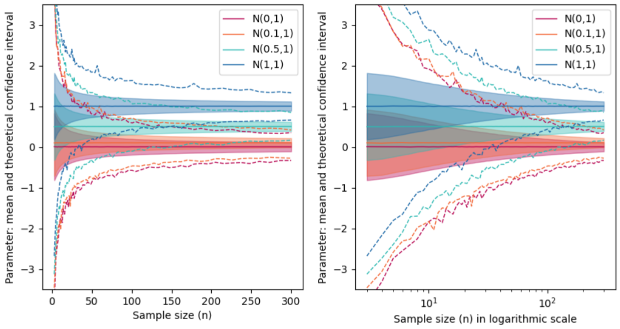

(a)

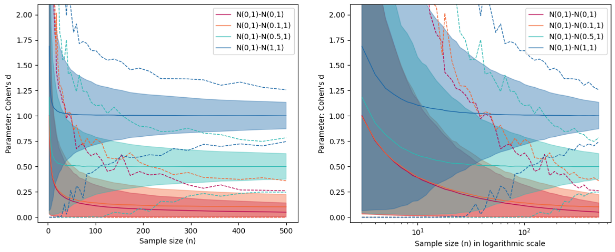

(b)

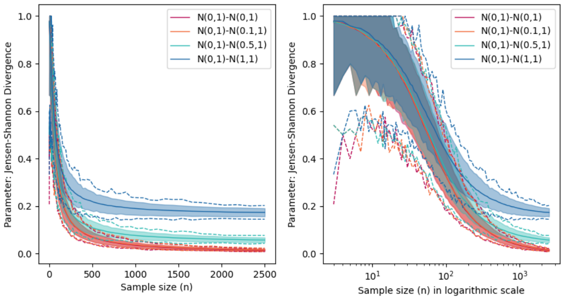

(c)

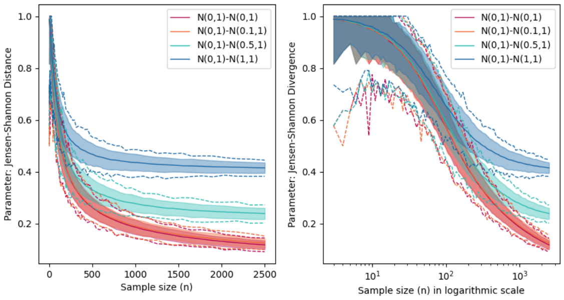

(d)

Fig. S1. (a) Estimated two-sided 95% confidence interval for the mean of different normal distributions with standard deviation of 1 and mean values of 0, 0.1, 0.5 or 1; (b) Cohen's d value, and (c) Jensen-Shannon divergence and (d) distance values between a normal distribution with standard deviation of 1 and mean value 0, and normal distributions with standard deviation of 1 and mean values of 0, 0.1, 0.5 or 1. In (a), for each fixed value of the sample size, 15000 confidence intervals for the mean of each normal distribution were calculated using the mathematical expression $x' \pm t\frac{s}{\sqrt{n}}$, where x' is the estimated mean value, t is the critical value of Student's t-distribution for a 0.025 of significance, s is the estimated variance and n is the sample size (filled area). The same procedure was followed in (b-d) with simulated datasets from the two compared normal distributions. The final confidence interval is obtained by clipping 95% of the values among the 15000 (filled area). The dashed lines show the maximum and minimum Jensen-Shannon divergence and distance values obtained for each sample size. The information is shown both in linear and logarithmic scale.

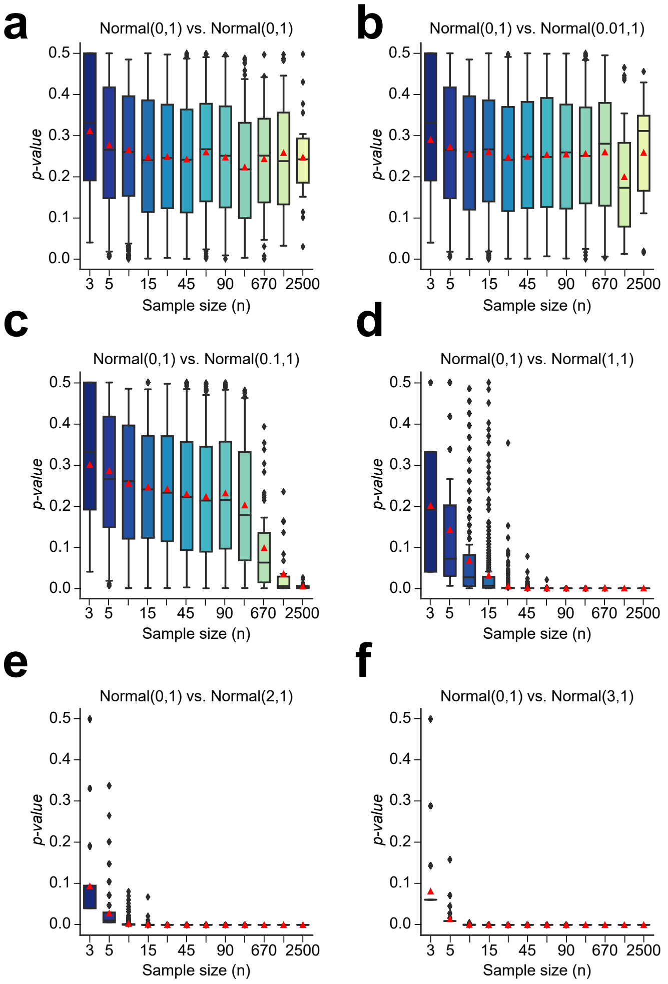

Fig. S2. Distribution of the *p-values* obtained when two normal distributions are compared. For each fixed value of the sample size ($3, 5, 10, 15, 30, 45, 60, 90, 200, 670$, 1750 and 2499 points), two normal distributions of that size are simulated and compared via the Mann-Whitney statistical test. This procedure is repeated multiple times. A normal distribution with a mean of 0 and a standard deviation of 1 is compared with a normal distribution of mean: (a) 0, (b) 0.01, (c) 0.1, (d) 1, (e) 2, and (f) 3 and a standard deviation of 1. When both normal distributions are almost the same, (a) and (b), the *p-value* follows a uniform distribution. Though, as long as both normal distributions get farther to each other, the distribution of *p-values* become closer to a normal distribution with a faster decay.

$$\mu_{\mathcal{U}} = \frac{n_1 n_2}{2}, \quad \sigma^2_{\mathcal{U}} = \frac{n_1 n_2 (n_1 + n_2 + 1)}{12}. \qquad [2]$$

Therefore, to estimate the *p-value* the standardized value of U, z, defined by

$$z = \frac{\mathrm{U} - \mu_{\mathrm{U}}}{\sigma_{\mathrm{U}}}. \qquad [3]$$

Replacing the values of U_i, $\mu_{\mathcal{U}}$ and $\sigma^2_{\mathcal{U}}$ in Eq. (3), we obtain

$$z = \sqrt{12}\left(\frac{n_1 n_2 + \frac{n_1(n_1+1)}{2} - R_1 - \frac{n_1 n_2}{2}}{\sqrt{n_1 n_2 (n_1 + n_2 + 1)}}\right). \qquad [4]$$

Since U is defined as the minimum of U_1 and U_2, we assume for simplicity that $\mathrm{U} = U_1$, but same results hold for $\mathrm{U} = U_2$. In the worst case scenario, when both datasets are identical and therefore the null hypothesis is true, $R_1 = R_2 = R$. Also, as n_i are assumed to be large enough, we can study the case $n_1 = n_2 = n$. Moreover, due to the hypothesized large sample size, R_i could be upper limited as

$$R \leq \sum_{i=1}^{n} i = \frac{n(n+1)}{2} \qquad \Longrightarrow \qquad z \geq \sqrt{12}\left(\frac{\frac{n^2}{2}}{\sqrt{n^2(2n+1)}}\right) = \frac{n\sqrt{3}}{\sqrt{(2n+1)}}. \qquad [5]$$

Finally, the value of z in the limit, when n tends to infinity, is also infinity

$$\lim_{n\to\infty} z \geq \lim_{n\to\infty} \frac{n\sqrt{3}}{\sqrt{(2n+1)}} \longrightarrow \infty \qquad \Longrightarrow \qquad \lim_{n\to\infty} z \longrightarrow \infty. \tag{6}$$

Therefore, the associated *p-value* is computed as the probability of a Gaussian distribution being greater than something that tends to infinity, which tends indeed to zero. That is to say, even when we assume that both datasets are equal, the result would be to regret the null hypothesis. Likewise, Student's t-test (5) suffers the same drawback. The statistic $\mathbf{t}$ is defined as follows

$$\mathbf{t} = \frac{\mu_1 - \mu_2}{\sqrt{\dfrac{S_1^2}{n_1} + \dfrac{S_2^2}{n_2}}}, \tag{7}$$

where μ_i and n_i correspond to the mean and sample size of the dataset $i \in \{1, 2\}$, and $S_i^2, i = 1, 2$ are the unbiased estimators of the variances of each of the samples. Once again, assuming that both n_i are large enough, $n_i = n$ is accepted; $\mathbf{t}$ is directly compared with the Student's t distribution and in the limit of n, $\mathbf{t}$ tends to infinity (as long as both mean values are not exactly the same). Thus, *p-value* tends to zero and the null hypothesis is rejected.

B. *p-values* as a function of the sample size. As it is shown in Fig. 2 and Fig. 5 of the main manuscript and Section A, the *p-value* depends on the size of the data being evaluated. While this is not a breakthrough, it is one of the pillars in this study. The fact that the *p-value* varies with n, allows us to assume that this relationship can be estimated through either a parametric or a non-parametric model. In this case, we fit a parametric model (i.e. the exponential function) to the *p-values* obtained empirically.

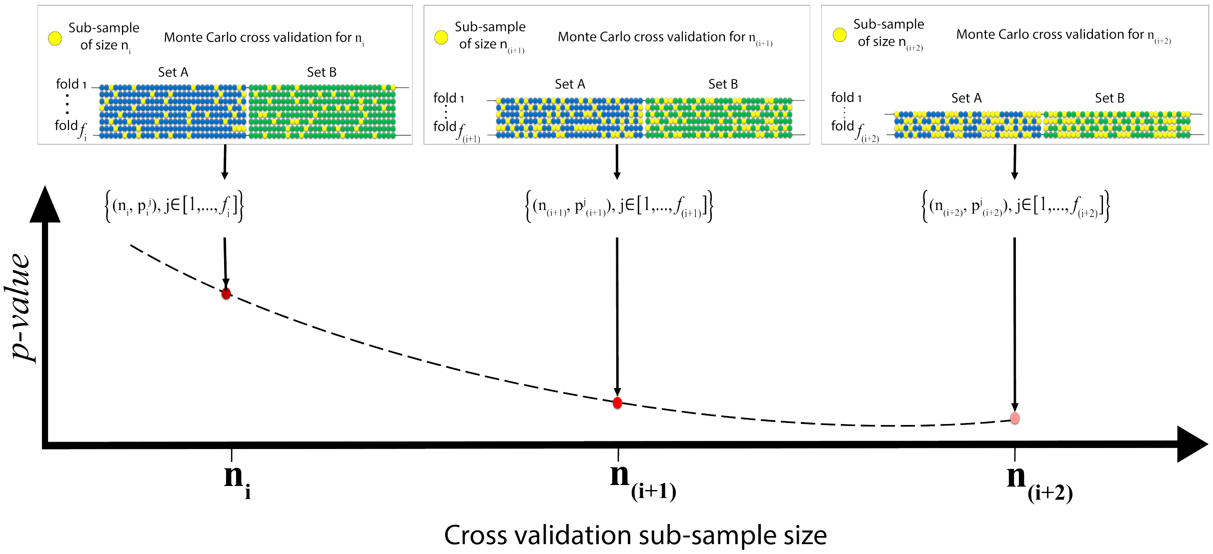

Fig. S3. Illustration of the work flow used for the estimation of *p-values* as a function ($p(n)$) of the sample size (n). For each possible value of n (n_i), Monte Carlo cross validation (MCCV) is performed f_i times. For each fold in the cross validation, two random sub-samples of size n_i are chosen from samples A and B (yellow spheres). Then, a statistical test is applied to obtain a *p-value* (p_i^j). The procedure is repeated f_i times; f_i depends on n_i as both samples A and B have to be covered. Thereby, f_i decreases ($f_i > f_{i+1} > f_{i+2}$) as long as n_i gets larger. This procedure is repeated for n_0, ..., n_{i+1},n_{i+2}, ... until the desired data size ($n_{i\to\infty}$) is reached.

C. Estimation of *p-values* with Monte Carlo cross validation method. We propose to model the *p-value* empirically as a data's size dependent function ($p(n)$) by Monte Carlo cross validation (MCCV) with replacement (6). This way, the effect of the sample bias on the *p-value* can be mitigated. The procedure followed to estimate of the *p-values* is illustrated in Figure S3 and the corresponding pseudocode in Algorithm 1. Notice that in Algorithm 1 we estimate $p(n)$ in two different ways, using either a locally weighted scatter plot smoothing (LOWESS) approximation (7) (p_L) or and exponential fit (p_e). The main reason to do this is that we use a standard curve fitting (LOWESS) to show that $p(n)$ can be expressed as an exponential function.

Aiming to compare two samples, S_A and S_B, and to determine if there exists statistically significant differences between them, the estimation of the *p-values* is done in pairs (i.e., two sub-samples, one extracted from S_A and the other from S_B, are compared each time). A range of values needs to be defined for both the sample size and the number of folds in MCCV. These are given by the grids $\mathcal{N}$ and $\mathcal{F}$, respectively.

The range for all possible sub-sample sizes (n) goes from 2 (n_0) to the smallest size of samples S_A and S_B (N_{max}). A grid covering all these values for large N_{max}, is computationally expensive and redundant. As the *p-value* tends to zero when $n \to \infty$,

Algorithm 1 *p-value* estimation

$N_{max} \leftarrow \min\{|S_A|, |(S_B|\}$

$\mathcal{N} \leftarrow \exp(grid[\log(n_0), \log(n_\infty), gridsize])$
$\mathcal{N} \leftarrow int(\mathcal{N})$

$a \leftarrow N_{max}/k_1 n_0$
$b \leftarrow k_2 N_{max}/n_\infty$
$\mathcal{F} \leftarrow \exp(grid[\log(a), \log(b), gridsize])$
$\mathcal{F} \leftarrow int(\mathcal{F})$

for i in $0 : \text{length}(\mathcal{N})$ **do**
  $n_i \leftarrow \mathcal{N}[i]$
  # Start Monte Carlo cross validation:
  for $f = 0 : \mathcal{F}[i]$ **do**
    $s_A \leftarrow \text{sample}(S_A, n_i)$
    $s_B \leftarrow \text{sample}(S_B, n_i)$
    $\mathcal{P}_i \leftarrow$ save the *p-value* of $test(s_A, s_B)$
  $\mathcal{P} \leftarrow$ save $mean(\mathcal{P}_i)$
  $\bar{\mathcal{P}} \leftarrow$ save $mean(\mathcal{P}_i)$
$p_L \leftarrow LOWESS(\bar{\mathcal{P}})$
$p_e \leftarrow exponential.fit(\mathcal{P})$

the most important information is condensed in the smallest values of n. So the grid $\mathcal{N}$ follows an exponential distribution from $n_0 = 2$. Similarly, a large enough upper-limit (n_∞) is chosen such that it ensures a fast computation ($n_\infty \ll N_{max}$) and the convergence to zero of $p(n)$. Hence, $\mathcal{N}$ is determined as

$$\mathcal{N} = \{n_i : n_i \in \exp\left(\mathcal{U}\left(\log(n_0), \log(n_\infty)\right)\right), n_i \in \mathbb{N}\}, \quad [8]$$

where $\mathcal{U}$ is the uniform distribution that goes from $\log(n_0)$ to $log(n_\infty)$. In MCCV, the number of folds can be extremely large when working with large datasets and a small partition. On the contrary, for a large partition size, the number of folds might decrease dramatically. To compensate for both situations, $\mathcal{F}$ is defined as given below

$$\mathcal{F} = \{f_i : f_i \in \exp\left(\mathcal{U}\left(a, b\right)\right), f_i \in \mathbb{N}\}, \text{ where } a = \log\left(\frac{N_{max}}{k_1 n_0}\right), b = \log\left(\frac{k_2 N_{max}}{n_\infty}\right), \quad [9]$$

$\mathcal{U}$ is the uniform distribution, k_1 controls the upper-limit on the number of folds for small sub-sample sizes, and k_2 controls the lower-limit for large sub-sample sizes. Note that the number of elements in $\mathcal{N}$ and $\mathcal{F}$ are the same.

Finally, for each n_i in $\mathcal{N}$, MCCV is applied to obtain the set of *p-values* defined as

$$\mathcal{P}_i = \left\{p_i^j, j \in [1, ..., f_i]\right\}, \quad [10]$$

being f_i the number of folds in $\mathcal{F}$ that corresponds with the sub-sample size n_i in $\mathcal{N}$.

D. Assessment of minimum data size needed for statistical significance (n_α). The estimation of the *p-value* function $p(n)$ supports the computation of the minimum data size needed to obtain statistically significant differences (n_α). This value is the solution to the equation

$$\alpha = ae^{-cn_\alpha}. \quad [11]$$

As explained in the main manuscript, the parameters a and c are the result of fitting an exponential function to the empirical values $\mathcal{P}_i$ in Equation 10. Therefore, there exists an intrinsic bias in the estimated values a and c. Additionally, the estimation of $p(n)$, and specially, its decay (parameter c) can be less precise when the data size is small. (See Figure 2*d* in the main manuscript), so n_α in Equation 11 can be biased.

For this reason, the calculation of a more conservative estimator, $\hat{n}_\alpha$, is strongly recommended

$$\hat{n}_\alpha = arg \min_{n_i} \left\{\left(\overline{p_i} + \sigma_{\overline{p_i}}\right) < \alpha\right\}, \quad [12]$$

where $\overline{p_i}$ represents the mean of $\mathcal{P}_i$ and $\sigma_{\overline{p_i}}$, the mean standard error (SEM), which is included to correct for the variability of the estimated *p-values*. Hence, the estimator of the theoretical value will always be slightly larger

$$\hat{n}_\alpha \geq n_\alpha.$$

However, $\hat{n}_\alpha$ can only be provided when the sample is large enough to cover those n values smaller or equal to $\hat{n}_\alpha$. For this reason, whenever the data is not large enough, the theoretical value n_α in Equation 11 is also given. In these cases, even if n_α and $\Theta_{\alpha,\gamma}$ might be slightly deviated, they still serve as an indicator of the existence of statistical significance.

3. Implementation details

We provide a ready-to-use Python code that implements the method explained above, outputs the decision index $\theta_{\alpha=0.05,\gamma} = 0$, $\hat{n}_{\alpha}$, a and c parameters, and generates the plots shown in the paper: https://github.com/BIIG-UC3M/pMoSS. Together with the implementation, different Jupyter Notebooks with demos and user guidelines are available, making this work accessible from both local machines and Google Colaboratory. The following pseudocode describes the process behind the algorithm. The description of MCCV (*monte_carlo_cross_validation*) is the one explained in Section C.

1. Initialization of all the parameters
2. **path**: location where the data is stored in the machine.
3. **file_name**: name of the file containing the numerical data (CSV or Excel).
4. **grid_size**: number of "n-values" to evaluate (size of $\mathcal{N}$-grid, Eq. 8.) (default: 200).
5. **n0**: minimum "n-value" to compute Monte Carlo cross-validation (default: 2).
6. **Nmax**: maximum "n-value" to compute Monte Carlo cross-validation (default: 1200).
7. **k**: this value prevents from having only one iteration for the highest "n-value", so
   *initial_iterations = np.log((m/n0)*initial_portion)*
   where m is the size of a group with less observations (default: 20).
8. **initial_portion**: this value prevents from having millions of iterations in *n0* so
   *initial_iterations = np.log((m/n0)*initial_portion)*
   where m is the size of the group with less observations (default: 1).
9. **alpha**: alpha for a 100(1-alpha)% statistical significance (default: 0.05).
10. **gamma**: the gamma value in the paper (default: $5e-06$).
11. **test**: statistical test to evaluate (default: 'MannWhitneyU').
12. **method**: method to estimate the *p-value* function (default: 'exponential').
13. Run the method
14. **pvalues, param, Theta** ← compute_diagnosis(file_name, path, gamma, alpha, grid_size, n0, Nmax, initial_portion, method, test)
15. compute_diagnosis{
    $\mathcal{P}$ ← monte_carlo_cross_validation(grid_size, n0, Nmax, k, initial_portion)
    p(n) ← exponential_fit()
    delta ← distance(p(n), alpha)
    Theta ← is(delta > 0)
    }
16. Save computed parameters
17. pvalues.to_csv('../data/morphology/aging_morphology_pvalues.csv', index = False)
18. Obtain the data, variables and name of the groups for the plot
19. data, variables, group_labels ← morphoparam(file_name, path = path)
20. Create all the comparison combinations from the list of groups
21. combination ← create_combination(group_labels)
22. Get a table with the results
23. table ← table_of_results(param, variables, combination)
24. Plot the results
25. composed_plot(data, pvalues, group_labels, combination, variables)

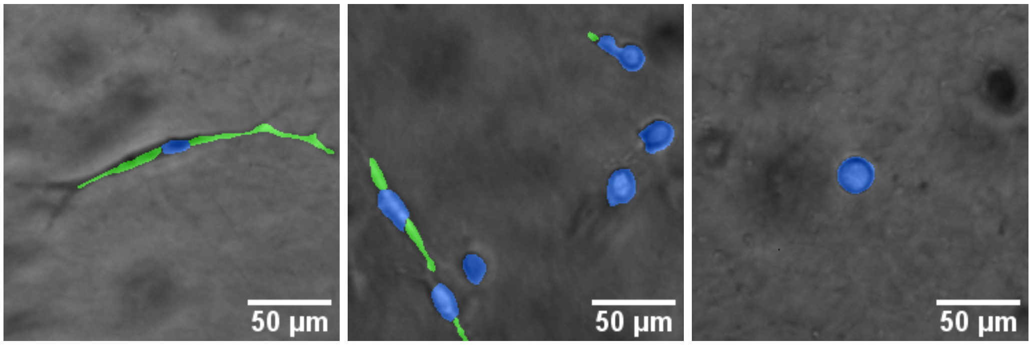

(a) (b) (c)

Fig. S4. Segmentation of phase contrast microscopy images of cancer cells (MDA-MB-231) embedded in a 3D collagen Type I matrix. Cell bodies are labeled in blue and cell protrusions in green. Images of (a) control cells and cells treated at (b) 1 nM and (c) 50 nM Taxol were acquired with a 10 x magnification objective.

4. Experimental data

A. Experiment 1**: Drug analysis on phase contrast microscopy data.** Image processing analysis provided the necessary information to distinguish the cellular body and protrusions of each cell in the videos (Figure S4). Hence, we got eight different measurements: cell body size (C_S), cell body perimeter (C_P), cell body roundness (C_R), cell with at least one protrusion (P_b), protrusion size (P_S), protrusion perimeter (P_P), protrusion length (P_L) and protrusion diameter (P_D). Each of the morphological measurements is given in microns. Table S1 contains the complete list of variables.

Table S1. List of computed variables. C: continuous variable. B: binary variable.

Cell body			Cell protrusions		
Feature	Name	Type	Feature	Name	Type
Area (μm^2)	C_S	Quantitative	Area (μm^2)	P_S	Quantitative
Perimeter (μm)	C_P	Quantitative	Perimeter (μm)	P_P	Quantitative
Roundness	C_R	Quantitative	Length (μm)	P_L	Quantitative
Protrusions	P_b	Binary	Diameter (μm)	P_D	Quantitative

In Figure S5, the distribution of the the variables used in the analysis of cellular shape is shown. The cellular body changes with the amount of Taxol used to treat cells. When they are treated at 50 nM Taxol, the cellular body is biger and more rounded (Figure S5a). Besides, this same treatment prevents cells from producing long and thick protrusions (Figure S5b).

None of the continuous variables presented in Table S1 follows a normal distribution, so the comparison was carried out by the Mann-Whitney U-test (1) for all the quantitative variables. P_b (Figure S5c) distinguished protruding cells (value one) from non-protruding ones (value zero). Therefore, it was analyzed by means of Pearson χ^2-test for categorical data (8).

As per the number of observations reported in Table S2 and following methodology guidelines, we set $\mathcal{P}_i$ with $n_0 = 2$ and $n_\infty = 2500$. $\mathcal{N}$ and $\mathcal{F}$ were set to have 190 points. The number of folds $\mathcal{F}$, Eq. 9, was computed using $k_1 = 1$, $k_2 = 20$ and $N_{max} = 11037$. These values were chosen to have a reasonable number of permutations for both small and large sample sizes (6000 permutations when $n_0 = 2$, and 90 when $n_{190} = 2500$, respectively). Table S3 contains the estimated coefficients a and c of the exponential curve (ae^{-cn}) for each of the variables we analyzed and each pair of comparisons (Control - 1 nM Taxol, Control - 50 nM Taxol, and 1 nM - 50 nM Taxol).

Treatment group	Cell body	Cell protrusions
Control	77700	45871
1 nM Taxol	74713	42798
50 nM Taxol	46162	11037

Table S2. Number of observations (cell body and their protrusions -if present-) per treatment group.

Figures S6, S7 and S8 show the shape of each of the exponential curves that result with the coefficients in Table S3. To determine whether Taxol has a significant effect in cell's morphology, $\Theta_{\alpha,\gamma}$ was chosen such that $\alpha = 0.05$ (95% of statistical significance) and $\gamma = 5 \cdot 10^{-6}$.

When comparing the control group and 1 nM Taxol, there are not practical differences in cell body morphology: the curve $p(n)$ of any cell body feature decreases slowly, i.e. $\hat{n}_\alpha$ and n_γ are large and $\Theta_{\alpha,\gamma} = 0$ (Table S3 and Figure S6a). On the other hand, cells at 50 nM Taxol have a significantly higher roundness index and bigger cellular body: when comparing control vs. 50 nM Taxol or 1 nM vs. 50 nM Taxol the curves corresponding to C_R and C_S decrease rapidly, i.e. $\hat{n}_\alpha$ and n_γ are small, and

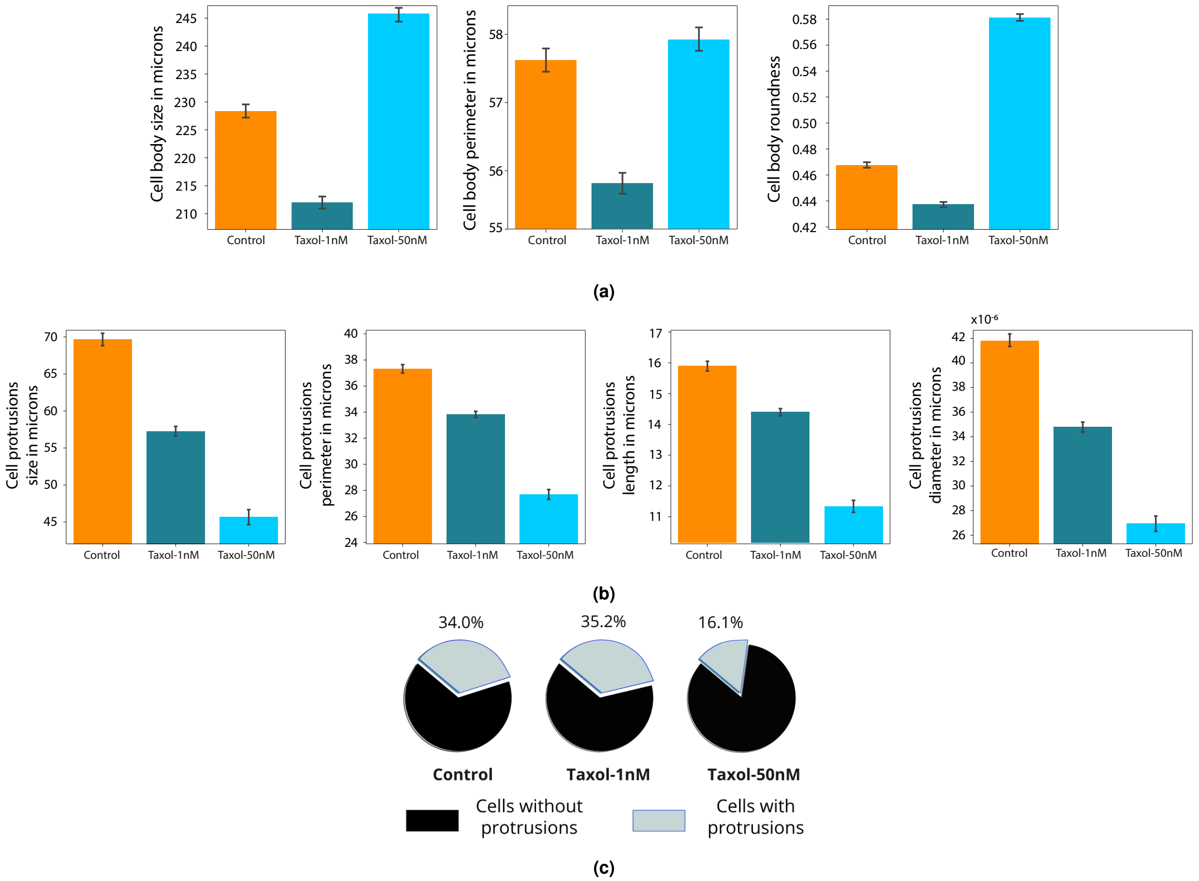

Fig. S5. Quantitative variables used to measure (a) cell bodies and (b) cellular protrusions morphology, and (c) the ratio of cells with and without protrusions for the three different treatment groups (control, 1 nM Taxol, 50 nM Taxol). Error bars in (a) and (b) correspond to the confidence interval at 99 %

$\Theta_{\alpha,\gamma} = 1$ (Table S3 and Figures S6b and S6c, respectively). For C_P, it is also possible to appreciate some differences when comparing 1 nM with 50 nM Taxol group, i.e. $\Theta_{\alpha,\gamma} = 1$ (Table S3). Namely, the blue curve shown in Figure S6c decreases faster than those in Figures S6a and S6b.

Similar results are obtained when the morphology of cellular protrusions is evaluated (Table S3 and Figure S7). While Taxol at 1 nM does not change their morphology ($\Theta_{\alpha,\gamma} = 0$ in Table S3 and Figure S7a), the effect of Taxol at 50 nM is much larger ($\Theta_{\alpha,\gamma} = 1$ in Table S3, Figures S6b and S6c) .

Usually, when a categorical variable such as P_b is analyzed, the input of a statistical test is a percentage rather than the raw data. Hence, when the binary variable P_b does not depend on the treatment, the $p(n)$ function shoots up, as for instance in Figure S8a. However, when there is some relationship or a meaningful effect of the treatment, $p(n)$ decreases and it is possible to analyze its decay, as in Figures S8b-c. With all, we can say that the formation of protrusions is inhibited when 50 nM Taxol are administered: there is a significant reduction in the number of cells that form protrusions and their protrusions are smaller (shorter and thinner) (S3, Figures S5b and S5c).

B. Experiment 2: Drug analysis on flow cytometry data. Flow cytometry is a technique that generates a large amount of data for each experiment. Consequently, any statistical test for groups comparison results in a vanishing *p-value*. To avoid that situation, practitioners tend to reduce the data to a single, representative measure for subject. For instance, Khoury *et al.* (9) acquired fluorescence intensity data from 6 different subjects and compute the median fluorescence intensity (MFI) for each of them. So, the statistical test is just applied on the 6 MFI values. However, our proposal of estimating the *p-value* as a function of the sample size enables to incorporate in the test the information given by the whole dataset and take into consideration the deviation and bias present in the data.

To illustrate the proposed procedure, we analyzed the flow cytometry data provided in (10) to determine the transcriptional changes induced by the *in vivo* exposure of human eosinophils to glucocorticoids. Khoury *et al.* (9) studied eosinophil surface proteins after being exposed to glucocorticoids and demonstrated that this exposure causes the apoptosis of human eosinophils (eosinopenia) once they migrate out of the blood circulation.

While they performed an extensive analysis, we have focused our study on the data related to the chemokine receptor gene CXCR4. In particular, the expression of CXCR4 on the surface of human eosinophils after being exposed for 2 hours to vehicle,

Variables	Cell body size (C_S)				Cell body perimeter (C_P)				Cell body roundness (C_R)				Cell with protrusions (P_b)			
Comparison	a	c	$\hat{n}_\alpha$	$\Theta_{\alpha,\gamma}$	a	c	$\hat{n}_\alpha$	$\Theta_{\alpha,\gamma}$	a	c	$\hat{n}_\alpha$	$\Theta_{\alpha,\gamma}$	a	c	$\hat{n}_\alpha$	$\Theta_{\alpha,\gamma}$
C - 1T	0.258	0.0026	670	**0**	0.258	0.0017	1160	**0**	0.259	0.0029	617	**0**	0.435	-0.0005	∞	**0**
C - 50T	0.263	0.0075	250	1	0.256	0.0014	1331	**0**	0.282	0.0400	47	1	0.198	0.0345	42	1
1T - 50T	0.272	0.0216	83	1	0.264	0.0072	257	1	0.292	0.0648	29	1	0.195	0.0351	41	1
Variables	Protrusions size (P_S)				Protrusions perimeter (P_P)				Protrusions length (P_L)				Protrusions diameter (P_D)			
Comparison	a	c	$\hat{n}_\alpha$	$\Theta_{\alpha,\gamma}$	a	c	$\hat{n}_\alpha$	$\Theta_{\alpha,\gamma}$	a	c	$\hat{n}_\alpha$	$\Theta_{\alpha,\gamma}$	a	c	$\hat{n}_\alpha$	$\Theta_{\alpha,\gamma}$
C - 1T	0.250	0.0031	563	1	0.248	0.0019	754	**0**	0.251	0.0011	1695	**0**	0.251	0.0023	707	**0**
C - 50T	0.246	0.0221	75	1	0.241	0.0276	58	1	0.250	0.0289	58	1	0.250	0.0248	68	1
1T - 50T	0.250	0.0100	170	1	0.256	0.0175	98	1	0.255	0.0211	80	1	0.247	0.0134	127	1

Table S3. Parameters of the exponential function ae^{-cn} and estimated minimum size $\hat{n}_\alpha$ for each of the analyzed variables. C: control, 1T: 1 nM Taxol and 50T: 50 nM Taxol. $a \in [0.195, 0.435]$, $c \in \left[-5 \cdot 10^{-4}, 0.0648\right]$, $\alpha = 0.05$ **and** $\gamma = 5 \cdot 10^{-6}$.

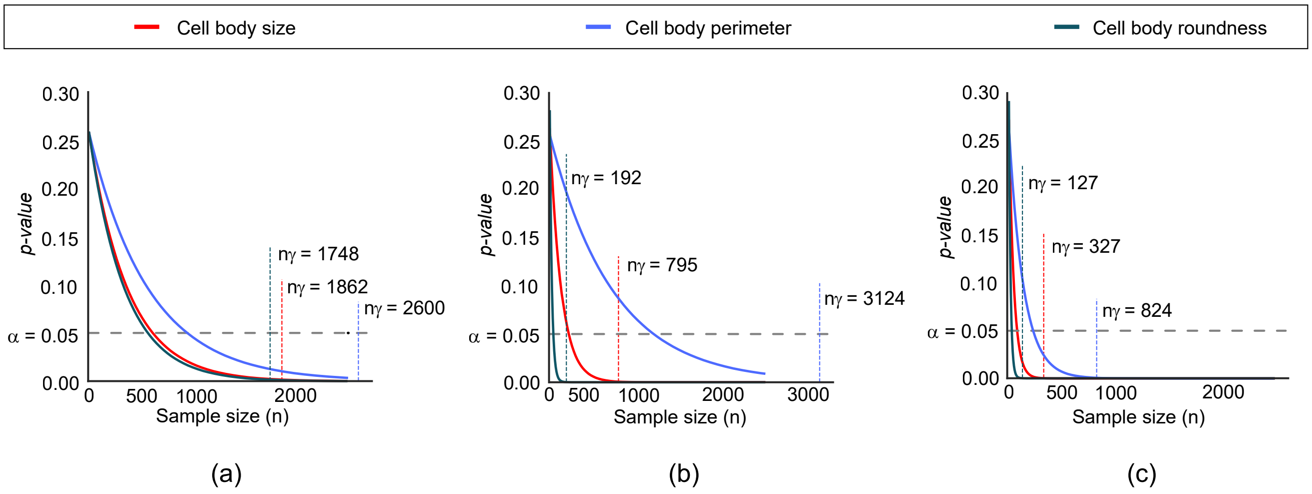

Fig. S6. Results obtained for cell body morphology when control, 1 nM Taxol and 50 nM Taxol groups are compared as (a) control vs. 1 nM Taxol, (b) control vs. 50 nM Taxol and (c) 1 nM vs. 50 nM Taxol. Vertical lines correspond to the convergence point n_γ with $\gamma = 5 \cdot 10^{-6}$.

20 mcg/dL and 200 mcg/dL of Methylprednisolone (MP). After filtering the raw data to discard noise and debris, we got clean distributions to analyze (Figure 2b, right). The *p-value* curves computed for pair group comparisons were the result of applying Mann-Whitney U statistical tests following the proposed procedure. Then, the exponential curves (ae^{-cn}) were fitted (Figure 2b, left). The parameters configuration was $n_0 = 2$, $\mathcal{N}$ and $\mathcal{F}$ had 200 points, $k_1 = 1$, $k_2 = 20$, $\alpha = 0.05$, and $\gamma = 5 \cdot 10^{-6}$. As the number of data points was lower than 1000, n_∞ and N_{max} where chosen to be the minimum number of points for each group pair being compared. The results are summarized in Table S4. Our results are similar to those in (9), in the sense that we also find a differential expression of CXCR4 when eosinophils are exposed to glucocorticoids.

C. Experiment 3: Cellular age characterization by means of biomolecular and biophysical properties. The data extracted from Phillip *et al.* in (11) was analysed with the following parameter configuration: for each of the stated variables, the data belonging to the group of 2 years-old was compared with the data from $\{3, 9, 16, 29, 35, 55, 65, 85, 92\}$ and $\{3, 9, 16, 29, 35, 55, 65, 85, 96\}$ years-old human donors to test cell motility and morphology, respectively. For each pair of groups, the distribution of the n-dependent *p-values* was obtained using the Mann-Whitney U statistical test. Then, the parameters of the exponential function (ae^{-cn}) were fitted. The parameter configuration was $n_0 = 2$, $\mathcal{N}$ and $\mathcal{F}$ had 200 points, $k_1 = 1$, $k_2 = 20$, $\alpha = 0.05$, and $\gamma = 5 \cdot 10^{-6}$. As the number of data points was lower than 1000, n_∞ and N_{max} where chosen to be the minimum number of points of each pair of groups being compared. The results for cell motility and cell morphology are summarized in Tables S5 and S6, respectively.

5. Test of robustness

The variability in the statistical significance of the results caused by the selection of the parameter γ and the grid sizes $\mathcal{N}$ and $\mathcal{F}$ are characterized in this section. The method is first tested using theoretical distributions and then, using the real data from Experiment 1.

A. Test of robustness on theoretical data. We simulated normal distributions to test the method in a theoretical scenario: $\mathcal{N}(0, 1)$ was compared with $\mathcal{N}(0.01, 1)$, $\mathcal{N}(0.1, 1)$, $\mathcal{N}(0.25, 1)$, $\mathcal{N}(0.5, 1)$, $\mathcal{N}(0.75, 1)$, $\mathcal{N}(1, 1)$, $\mathcal{N}(2, 1)$ and $\mathcal{N}(3, 1)$. For the most

Measures	CXCR4 surface expression				
Comparison	a	c	$\hat{n}_\alpha$	n_α	$\Theta_{\alpha,\gamma}$
Vehicle - MP 20 mcg/dL	0.286	0.048	40	36	1
Vehicle - MP 200 mcg/dL	0.286	0.049	37	35	1
MP 20 mcg/dL - MP 200 mcg/dL	0.256	$1.69 \cdot 10^{-4}$	-	9680	0

Table S4. Parameters of the exponential function ae^{-cn} for the differential expression of CXCR4, theoretical minimum size (n_α) and its estimator ($\hat{n}_\alpha$) for a 95% ($\alpha = 0.05$) of statistical significance, and decision index $\Theta_{\alpha,\gamma}$, for $\gamma = 5 \cdot 10^{-6}$. MP: Methylprednisolone

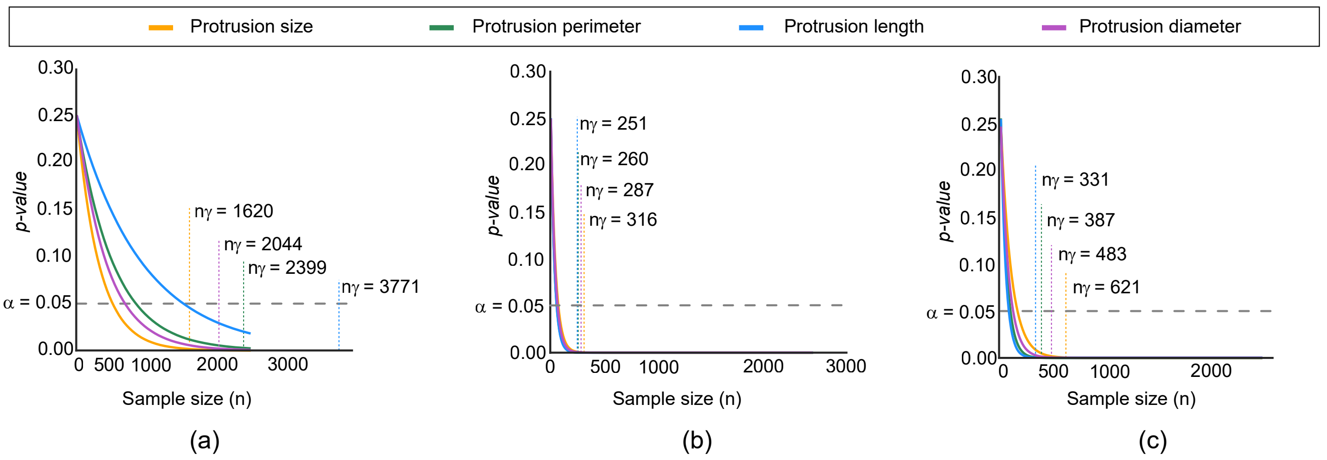

Fig. S7. Graphical illustration of the results obtained for cell protrusions morphology when (a) control and 1 nM Taxol, (b) control and 50 nM Taxol and (c) 1 nM Taxol and 50 nM Taxol groups are compared. Vertical lines correspond to the convergence point n_γ with $\gamma = 5 \cdot 10^{-6}$.

similar cases such as $\mathcal{N}(0,1)$ vs. $\mathcal{N}(0.01,1)$, or $\mathcal{N}(0,1)$ vs. $\mathcal{N}(0.1,1)$, it is expected to obtain $\Theta_{\alpha,\gamma} = 0$. While for the most different distributions such as $\mathcal{N}(0,1)$ vs. $\mathcal{N}(2,1)$, or $\mathcal{N}(0,1)$ vs. $\mathcal{N}(3,1)$, $\Theta_{\alpha,\gamma} = 1$.

Theoretically, an optimal grid $\mathcal{N}$ would be the one that covers the values from $n_0 = 2$ to $n_\infty = N_{max}$. This set up entails an extremely large amount of computations, while it suffices a value $n_\infty \approx 1000$ to understand what is the trend of the data. If $p(n)$ converges to zero when $n > 1000$, then it can be assumed that $p(n)$ does not represent a statistical significant case. Hence, $n_\infty = 2500$ is large enough for the implementation of the method. As the *p-values* for very small samples are especially unstable and small samples are not representative of any real scenario, the minimum value n_0 can be increased. The number of permutations for each n_i can be decreased as well: while the amount of data to analyze may be infinite, it is enough to study a certain limited number of different data subsamples to approach a realistic scenario. Hence, to test the robustness of the proposed method, we set grids $\mathcal{N}$ and $\mathcal{F}$ using $n_0 = 20$, $n_\infty = 2500$, $N_{max} = 10000$, $k_1 = 1$ and $k_2 = 20$ in Equations 8 and 9, respectively. Both $\mathcal{N}$ and $\mathcal{F}$ were configured to have a size of 200 points. Thus, MCCV is repeated 200 times. See Figure S3 for the workflow.

With this grid parameters, for γ in the set $\{2.5 \cdot 10^{-6}, 5 \cdot 10^{-6}, 5 \cdot 10^{-5}, 5 \cdot 10^{-4}\}$, we run the pipeline to evaluate the effect of γ value on the rejection of the null hypothesis of the Mann-Whitney U statistical test (Table S7). The results obtained help to assess the most suitable γ value. Specifically, the decision about γ relies on the result obtained for the comparison between $\mathcal{N}(0,1)$ and $\mathcal{N}(0.25,1)$: while the distance $\delta_{\alpha,\gamma}$ for $\gamma = 5 \cdot 10^{-6}$ and $\gamma = 5 \cdot 10^{-5}$ expresses the same ($\delta_{\alpha,\gamma} = \pm 5.84$), the minimum data size needed to observe statistically significance differences is low enough as to reject the null hypothesis, i.e. $\hat{n}_\alpha = 186$ and $\Theta_{\alpha,\gamma} = 1$. Hence, the value chosen for the following simulations and for the real data is $\gamma = 5 \cdot 10^{-6}$. (Table S7).

To test the computational limitations of the method, we evaluated the value $\Theta_{0.05,5\cdot10^{-6}}$ reducing $\mathcal{N}$ and $\mathcal{F}$: $\mathcal{N}$ was chosen to be a grid of size 10, 20, 50, 100, 150 or 200 points and the values in $\mathcal{F}$ were reduced by a factor of 1/2, 1/3, 1/5 and 1/10 (i.e., each of the values in the original $\mathcal{F}$ was multiplied by this fraction). The experiment was repeated 100 times on each of the setups, so the probability of obtaining exactly the same $\Theta_{0.05,5\cdot10^{-6}}$ (Table S7) and the stability of the method could be evaluated. The information given in Table S8 lets the assessment of (1) the size of $\mathcal{N}$ and (2) the number of folds in $\mathcal{F}$. In most cases, the probability obtained was 100%, which shows that the final results are very stable. When $N(0,1)$ and $N(0.5,1)$ were compared with small grid parameters, this probability decreased slightly to $89-96\%$ (Table S8). In conclusion, the number of computations could be considerably reduced, for the example, to $\mathcal{N} = 50$ and $\mathcal{F} = 0.2\mathcal{F}$).

B. Test of robustness on real data.

B.1. The* p-value *can be estimated by an exponential function. Repeating the procedure followed with simulation of normal distributions, we verify that the condition for $p(n)$ being exponential is satisfied again: in Figures S9a-c all LOWESS fittings have exponential shapes, and in Figures S9d-f the ratio $p'(n)/p(n)$ of LOWESS fits are constant.

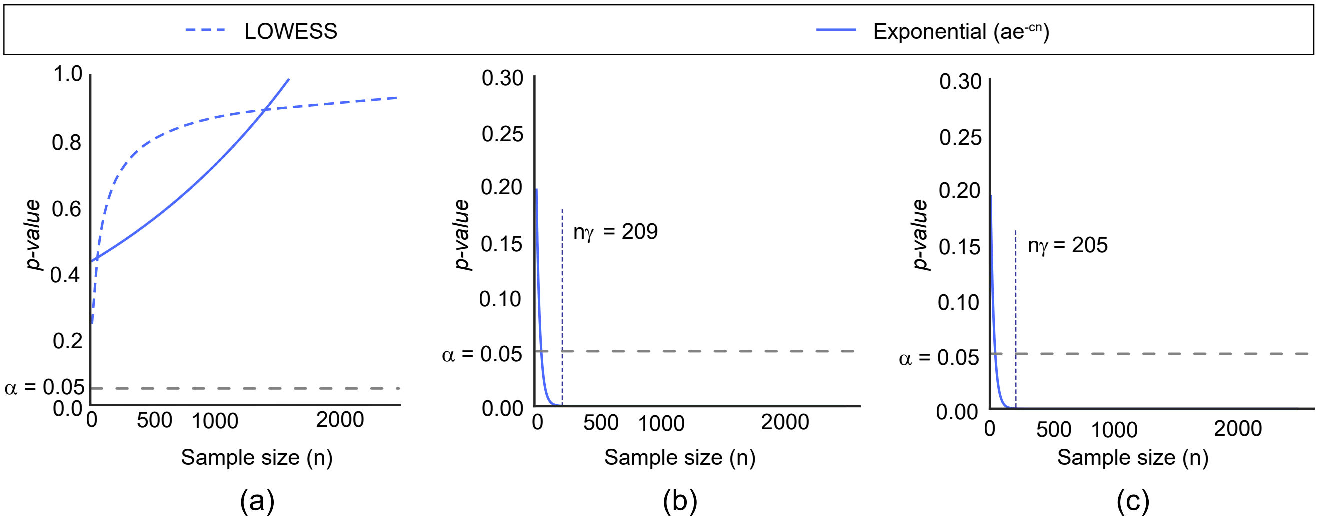

Fig. S8. Estimation of $p(n)$ with χ^2 test for contingency tables when comparing cells (not) having at least one protrusion (binary variable cell with protrusions: P_b) for (a) control and 1 nM Taxol groups, (b) control and 50 nM Taxol groups, and (c) 1 nM and 50 nM Taxol groups. In the leftmost plot, both locally weighted scatter plot smoothing (LOWESS) and exponential fit of estimated *p-values* are shown. Vertical lines correspond to the convergence point n_γ with $\gamma = 5 \cdot 10^{-6}$.

B.2. Robustness of the convergence threshold and required computational load. The distribution of real data is more complex than the typical Gaussian distributions due to the presence of noise, large deviation of the data or leverage points. Following the same procedure as in Section A, we tested the reliability of the proposed method using the data we extracted from the microscopy images (Experiment 1). We evaluated both, the effect of varying the convergence threshold γ and the required computational load. Looking at Tables S3 and S9, it can be appreciated once again that $\gamma = 5 \cdot 10^{-6}$ is a good value for the convergence threshold. Smaller values of γ result in the rejection of the null hypothesis for cases in which $\hat{n}_\alpha > 1000$ as cellular protrusions length. Similarly, when $\gamma = 5 \cdot 10^{-5}$, there are cases as cell body roundness for which $\hat{n}_\alpha < 100$ and $\Theta_{\alpha,\gamma} = 0$. Therefore, once again, $\gamma = 5 \cdot 10^{-6}$ seems to be an appropriate value to measure statistical significance at $\alpha = 0.05$ significance level (Table S9).

When the grid $\mathcal{N}$ is large enough and $\mathcal{F}$ has large numbers, the result is completely stable (Table S10). However, when these values are dramatically reduced (for example, $\mathcal{N} = 10$, $\mathcal{F} = 0.02\mathcal{F}_0$, $\mathcal{F} = 0.01\mathcal{F}_0$ where $\mathcal{F}_0$ is the initial grid that we used and the one we compare the results with), the reproducibility of the results may degrade. This fact is specially evident when the variables are noisy as in the case of those that measure cellular protrusions morphology (P_S, P_P, P_L and P_D), being the noisier the protrusions size and perimeter. In summary, while large grid parameters ensure stable results, with the information given in Tables S8 and S10, it seems reasonable to reduce the number of computations to $\mathcal{N} \geq 50$ and $\mathcal{F} \geq 0.2\mathcal{F}_0$.

Measures	MSD-6min					MSD-60 min					Persistence primary axis				
Comparison	a	c	$\hat{n}_\alpha$	n_α	$\Theta_{\alpha,\gamma}$	a	c	$\hat{n}_\alpha$		$\Theta_{\alpha,\gamma}$	a	c	$\hat{n}_\alpha$	n_α	$\Theta_{\alpha,\gamma}$
A02 - A03	0.296	0.038	46	46	1	0.276	0.009	-	187	1	0.287	0.012	-	145	1
A02 - A09	0.270	0.001	-	1333	0	0.295	0.000	-	$4.72 \cdot 10^{17}$	0	0.276	0.002	-	896	0
A02 - A16	0.289	0.014	-	125	1	0.262	0.001	-	1955	0	0.310	0.084	23	21	1
A02 - A29	0.394	0.217	12	9	1	0.357	0.161	13	12	1	0.287	0.013	-	137	1
A02 - A35	0.315	0.155	12	11	1	0.335	0.123	18	15	1	0.274	0.002	-	752	0
A02 - A55	0.286	0.018	-	99	1	0.283	0.032	52	54	1	0.286	0.000	-	$4.99 \cdot 10^{15}$	0
A02 - A65	0.306	0.032	48	56	1	0.345	0.113	15	17	1	0.330	0.097	19	19	1
A02 - A85	0.282	0.008	-	229	1	0.303	0.040	46	45	1	0.315	0.050	34	36	1
A02 - A92	0.345	0.189	12	10	1	0.386	0.228	10	8	1	0.331	0.079	27	24	1

Measures	Persistence secondary axis					Diffusivity primary axis					Diffusivity secondary axis				
Comparison	a	c	$\hat{n}_\alpha$	n_α	$\Theta_{\alpha,\gamma}$	a	c	$\hat{n}_\alpha$	n_α	$\Theta_{\alpha,\gamma}$	a	c	$\hat{n}_\alpha$	n_α	$\Theta_{\alpha,\gamma}$
A02 - A03	0.276	0.001	-	1399	0	0.301	0.000	-	1.47E+17	0	0.290	0.034	52	51	1
A02 - A09	0.266	0.001	-	3023	0	0.275	0.006	-	289	1	0.279	0.051	32	33	1
A02 - A16	0.295	0.069	27	25	1	0.293	0.030	56	58	1	0.268	0.004	-	443	0
A02 - A29	0.301	0.019	-	94	1	0.335	0.078	27	24	1	0.354	0.160	15	12	1
A02 - A35	0.281	0.014	-	126	1	0.312	0.061	30	29	1	0.312	0.117	19	15	1
A02 - A55	0.284	0.015	-	116	1	0.285	0.016	-	109	1	0.284	0.008	-	213	1
A02 - A65	0.294	0.030	-	58	1	0.327	0.086	24	21	1	0.312	0.100	15	18	1
A02 - A85	0.287	0.027	54	65	1	0.304	0.051	33	35	1	0.269	0.004	-	397	1
A02 - A92	0.293	0.016	-	112	1	0.380	0.217	11	9	1	0.295	0.025	-	71	1

Measures	Total diffusivity					Anisotropy				
Comparison	a	c	$\hat{n}_\alpha$	n_α	$\Theta_{\alpha,\gamma}$	a	c	$\hat{n}_\alpha$	n_α	$\Theta_{\alpha,\gamma}$
A02 - A03	0.284	0.000	-	$8.70 \cdot 10^{18}$	0	0.291	0.024	69	72	1
A02 - A09	0.280	0.000	-	$1.68 \cdot 10^{19}$	0	0.307	0.075	26	24	1
A02 - A16	0.297	0.036	40	48	1	0.293	0.000	-	$1.16 \cdot 10^{15}$	0
A02 - A29	0.360	0.122	17	16	1	0.290	0.016	-	106	1
A02 - A35	0.330	0.093	22	20	1	0.281	0.014	-	120	1
A02 - A55	0.276	0.006	-	269	1	0.309	0.043	40	42	1
A02 - A65	0.330	0.105	19	18	1	0.266	0.000	-	35475	0
A02 - A85	0.306	0.052	33	34	1	0.293	0.015	-	120	1
A02 - A92	0.379	0.201	11	10	1	0.359	0.104	20	19	1

Table S5. Parameters of the exponential function ae^{-cn} for cell motility, theoretical minimum size (n_α) and estimated one ($\hat{n}_\alpha$) for a 95% ($\alpha = 0.05$) of statistical significance, and decision index $\Theta_{\alpha,\gamma}$, for $\gamma = 5 \cdot 10^{-6}$.

References

1. Mann HB, Whitney DR (1947) On a Test of Whether one of Two Random Variables is Stochastically Larger than the Other. *Ann. Math. Stat.* 18(1):50–60.
2. Altman N, Krzywinski M (2017) P values and the search for significance. *Nat. Methods* 14(1):3–4.
3. Krzywinski M, Altman N (2013) Significance, P values and t-tests. *Nat. Methods* 10(11):1041–1042.
4. Krzywinski M, Altman N (2013) Error bars. *Nat. Methods* 10(10):921–922.
5. Student (1908) The Probable Error of a Mean. *Biometrika* 6(1):1.
6. Xu QS, Liang YZ (2001) Monte Carlo cross validation. *Chemom. Intell. Lab. Syst.* 56(1):1–11.
7. Cleveland WS (1979) Robust Locally Weighted Regression and Smoothing Scatterplots. *J. Am. Stat. Assoc.* 74(368):829–836.
8. Pearson K (1900) X. On the criterion that a given system of deviations from the probable in the case of a correlated system of variables is such that it can be reasonably supposed to have arisen from random sampling. *London, Edinburgh, Dublin Philos. Mag. J. Sci.* 50(302):157–175.
9. Khoury P, et al. (2018) Glucocorticoid-induced eosinopenia in humans can be linked to early transcriptional events. *Allergy* 73(10):2076–2079.
10. Gadkari M, et al. (2018) Transcript- and protein-level analyses of the response of human eosinophils to glucocorticoids. *Sci. Data* 5:180275.
11. Phillip JM, et al. (2017) Biophysical and biomolecular determination of cellular age in humans. *Nat. Biomed. Eng.* 1(7):0093.

Measures	Size (pixels)					Perimeter (pixels)					Long axis length (pixels)				
Comparison	a	c	$\hat{n}_\alpha$	n_α	$\Theta_{\alpha,\gamma}$	a	c	$\hat{n}_\alpha$	n_α	$\Theta_{\alpha,\gamma}$	a	c	$\hat{n}_\alpha$	n_α	$\Theta_{\alpha,\gamma}$
A02 - A03	0.267	0.002	509	688	0	0.271	0.009	203	183	1	0.276	0.022	76	76	1
A02 - A09	0.264	0.002	-	923	0	0.260	0.000	-	22578	0	0.274	0.008	-	201	1
A02 - A16	0.320	0.149	13	12	1	0.340	0.210	10	9	1	0.477	0.459	6	4	1
A02 - A29	0.312	0.129	16	14	1	0.313	0.121	16	15	1	0.325	0.144	14	12	1
A02 - A35	0.311	0.106	19	17	1	0.295	0.084	21	21	1	0.316	0.133	16	13	1
A02 - A55	0.313	0.112	18	16	1	0.323	0.136	13	13	1	0.364	0.243	10	8	1
A02 - A65	0.326	0.137	16	13	1	0.373	0.270	8	7	1	0.401	0.335	7	6	1
A02 - A85	0.330	0.167	13	11	1	0.431	0.401	6	5	1	0.442	0.403	6	5	1
A02 - A96	0.351	0.252	9	7	1	0.370	0.320	7	6	1	0.422	0.421	5	5	1
Measures	Short axis length (pixels)					Orientation					Solidity				
Comparison	a	c	$\hat{n}_\alpha$	n_α	$\Theta_{\alpha,\gamma}$	a	c	$\hat{n}_\alpha$	n_α	$\Theta_{\alpha,\gamma}$	a	c	$\hat{n}_\alpha$	n_α	$\Theta_{\alpha,\gamma}$
A02 - A03	0.267	0.005	284	330	1	0.265	0.000	-	$2.38 \cdot 10^{18}$	0	0.273	0.013	124	126	1
A02 - A09	0.263	0.000	-	3801	0	0.267	0.000	-	$1.09 \cdot 10^{16}$	0	0.280	0.026	69	65	1
A02 - A16	0.264	0.005	321	345	1	0.262	0.001	-	$1.41 \cdot 10^{03}$	0	0.266	0.003	386	485	0
A02 - A29	0.284	0.040	48	43	1	0.262	0.000	-	$1.53 \cdot 10^{14}$	0	0.276	0.025	74	68	1
A02 - A35	0.272	0.011	159	160	1	0.265	0.000	-	$4.60 \cdot 10^{17}$	0	0.318	0.125	16	14	1
A02 - A55	0.260	0.000	-	6616	0	0.265	0.000	-	$1.65 \cdot 10^{20}$	0	0.267	0.005	267	319	1
A02 - A65	0.291	0.057	34	30	1	0.262	0.001	-	$3.20 \cdot 10^{03}$	0	0.275	0.015	117	112	1
A02 - A85	0.311	0.103	20	17	1	0.265	0.000	-	$8.27 \cdot 10^{16}$	0	0.321	0.177	11	10	1
A02 - A96	0.295	0.069	27	25	1	0.261	0.000	-	$1.13 \cdot 10^{16}$	0	0.298	0.077	26	23	1
Measures	Equivalent diameter (pixels)					Aspect ratio					Circularity				
Comparison	a	c	$\hat{n}_\alpha$	n_α	$\Theta_{\alpha,\gamma}$	a	c	$\hat{n}_\alpha$	n_α	$\Theta_{\alpha,\gamma}$	a	c	$\hat{n}_\alpha$	n_α	$\Theta_{\alpha,\gamma}$
A02 - A03	0.267	0.002	509	688	0	0.282	0.027	74	63	1	0.277	0.022	78	77	1
A02 - A09	0.264	0.002	-	923	0	0.277	0.016	103	104	1	0.261	0.000	-	$1.33 \cdot 10^{13}$	0
A02 - A16	0.320	0.149	13	12	1	0.384	0.327	7	6	1	0.319	0.162	12	11	1
A02 - A29	0.312	0.129	16	14	1	0.275	0.019	86	90	1	0.260	0.001	-	2313	0
A02 - A35	0.311	0.106	19	17	1	0.281	0.042	44	40	1	0.259	0.000	-	$1.22 \cdot 10^{15}$	0
A02 - A55	0.313	0.112	18	16	1	0.318	0.154	13	12	1	0.289	0.056	36	31	1
A02 - A65	0.326	0.137	16	13	1	0.306	0.094	20	19	1	0.313	0.113	18	16	1
A02 - A85	0.330	0.167	13	11	1	0.290	0.068	27	25	1	0.338	0.238	9	8	1
A02 - A96	0.351	0.252	9	7	1	0.326	0.149	13	12	1	0.337	0.187	11	10	1
Measures	Roundness														
Comparison	a	c	$\hat{n}_\alpha$	n_α	$\Theta_{\alpha,\gamma}$										
A02 - A03	0.277	0.022	78	77	1										
A02 - A09	0.261	0.000	-	$1.33 \cdot 10^{13}$	0										
A02 - A16	0.319	0.162	12	11	1										
A02 - A29	0.260	0.001	-	2313	0										
A02 - A35	0.259	0.000	-	$1.2 \cdot 10^{15}$	0										
A02 - A55	0.289	0.056	36	31	1										
A02 - A65	0.313	0.113	18	16	1										
A02 - A85	0.338	0.238	9	8	1										
A02 - A96	0.337	0.187	11	10	1										

Table S6. Parameters of the exponential function ae^{-cn} for cell nuclei morphology measures, theoretical minimum size (n_α) and estimated one ($\hat{n}_\alpha$) for a 95% ($\alpha = 0.05$) of statistical significance, and decision index $\Theta_{\alpha,\gamma}$, for $\gamma = 5 \cdot 10^{-6}$.

γ	$2.5 \cdot 10^{-6}$		$5 \cdot 10^{-6}$		$5 \cdot 10^{-5}$		$5 \cdot 10^{-4}$		
Comparison	$\Theta_{\alpha,\gamma}$	$\delta_{\alpha,\gamma}$	$\Theta_{\alpha,\gamma}$	$\delta_{\alpha,\gamma}$	$\Theta_{\alpha,\gamma}$	$\delta_{\alpha,\gamma}$	$\Theta_{\alpha,\gamma}$	$\delta_{\alpha,\gamma}$	$\hat{n}_\alpha$
$\mathcal{N}(0,1)$ - $\mathcal{N}(0,1)$	0	-490.25	0	-490.25	0	-490.25	0	-490.25	0
$\mathcal{N}(0,1)$ - $\mathcal{N}(0.01,1)$	0	-3576	0	-488.25	0	-488.25	0	-488.25	0
$\mathcal{N}(0,1)$ - $\mathcal{N}(0.1,1)$	1	0.782	0	-21.922	0	-79.938	0	-42.375	1237
$\mathcal{N}(0,1)$ - $\mathcal{N}(0.25,1)$	1	9.523	1	5.848	0	-5.840	0	-12.945	186
$\mathcal{N}(0,1)$ - $\mathcal{N}(0.5,1)$	1	0	1	0	1	0	1	0	45
$\mathcal{N}(0,1)$ - $\mathcal{N}(0.75,1)$	1	0	1	0	1	0	1	0	22
$\mathcal{N}(0,1)$ - $\mathcal{N}(1,1)$	1	0	1	0	1	0	1	0	0
$\mathcal{N}(0,1)$ - $\mathcal{N}(1.5,1)$	1	0	1	0	1	0	1	0	0
$\mathcal{N}(0,1)$ - $\mathcal{N}(2,1)$	1	0	1	0	1	0	1	0	0
$\mathcal{N}(0,1)$ - $\mathcal{N}(2.5,1)$	1	0	1	0	1	0	1	0	0
$\mathcal{N}(0,1)$ - $\mathcal{N}(3,1)$	1	0	1	0	1	0	1	0	0

Table S7. Table of decision index $\Theta_{\alpha,\gamma}$, difference $\delta_{\alpha,\gamma} = A_{\alpha_\gamma} - A_{p(n_\gamma)}$ and estimated minimum size ($\hat{n}_\alpha$) for $\alpha = 0.05$ and $\gamma = 2.5 \cdot 10^{-6}, 5 \cdot 10^{-6}, 5 \cdot 10^{-5}, 5 \cdot 10^{-4}$.

		$\mathcal{N}$ grid size								$\mathcal{N}$ grid size					
Comparison	$\mathcal{F}$ reduction	10	20	50	100	150	200	Comparison	$\mathcal{F}$ reduction	10	20	50	100	150	200
$\mathcal{N}(0,1)$ - $\mathcal{N}(0,1)$	0.1	100	100	100	100	100	100	$\mathcal{N}(0,1)$ - $\mathcal{N}(0.75,1)$	0.1	100	100	100	100	100	100
	0.2	100	100	100	100	100	100		0.2	100	100	100	100	100	100
	1/3	100	100	100	100	100	100		1/3	100	100	100	100	100	100
	0.5	100	100	100	100	100	100		0.5	100	100	100	100	100	100
	1	100	100	100	100	100	100		1	100	100	100	100	100	100
$\mathcal{N}(0,1)$ - $\mathcal{N}(0.01,1)$	0.1	100	100	100	100	100	100	$\mathcal{N}(0,1)$ - $\mathcal{N}(1,1)$	0.1	100	100	100	100	100	100
	0.2	100	100	100	100	100	100		0.2	100	100	100	100	100	100
	1/3	100	100	100	100	100	100		1/3	100	100	100	100	100	100
	0.5	100	100	100	100	100	100		0.5	100	100	100	100	100	100
	1	100	100	100	100	100	100		1	100	100	100	100	100	100
$\mathcal{N}(0,1)$ - $\mathcal{N}(0.1,1)$	0.1	99	100	100	100	100	100	$\mathcal{N}(0,1)$ - $\mathcal{N}(1.5,1)$	0.1	100	100	100	100	100	100
	0.2	100	100	100	100	100	100		0.2	100	100	100	100	100	100
	1/3	100	100	100	100	100	100		1/3	100	100	100	100	100	100
	0.5	100	100	100	100	100	100		0.5	100	100	100	100	100	100
	1	100	100	100	100	100	100		1	100	100	100	100	100	100
$\mathcal{N}(0,1)$ - $\mathcal{N}(0.25,1)$	0.1	100	100	100	100	100	100	$\mathcal{N}(0,1)$ - $\mathcal{N}(2,1)$	0.1	100	100	100	100	100	100
	0.2	100	100	100	100	100	100		0.2	100	100	100	100	100	100
	1/3	100	100	100	100	100	100		1/3	100	100	100	100	100	100
	0.5	100	100	100	100	100	100		0.5	100	100	100	100	100	100
	1	100	100	100	100	100	100		1	100	100	100	100	100	100
$\mathcal{N}(0,1)$ - $\mathcal{N}(0.5,1)$	0.1	89	95	99	99	100	100	$\mathcal{N}(0,1)$ - $\mathcal{N}(3,1)$	0.1	100	100	100	100	100	100
	0.2	96	99	100	100	100	100		0.2	100	100	100	100	100	100
	1/3	100	99	100	100	100	100		1/3	100	100	100	100	100	100
	0.5	99	100	100	100	100	100		0.5	100	100	100	100	100	100
	1	100	100	100	100	100	100		1	100	100	100	100	100	100

Table S8. Table of results for different sizes of $\mathcal{N}$ and $\mathcal{F}$ grids. Each value represents the probability (%) of obtaining the same decision index $\Theta_{0.05,5\cdot10^{-6}}$ as the one shown in Table S7.

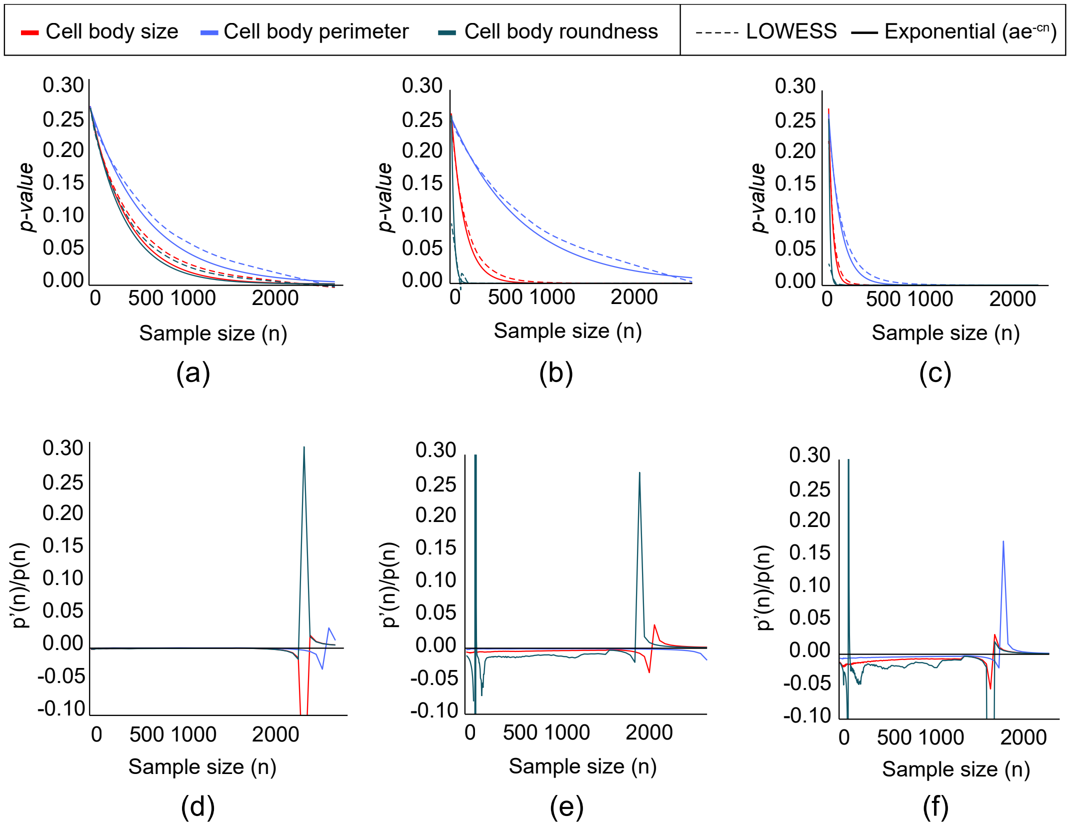

Fig. S9. Curve fitting. The function $p(n)$ is modelled to compare the variables obtained from the three groups in our experiment. Cell body size, perimeter and roundness of three different cell groups where compared by means of the Mann-Whitney U statistical test and Monte Carlo cross validation: (a) and (d) control cells versus cells treated with Taxol at 1 nM; (b) and (e) control cells versus cells treated with Taxol at 50 nM and (c) and (f) cells treated with Taxol at 1 nM and 50 nM. To each of the mean *p-value* sets (i.e., the output of Monte Carlo cross validation), a locally weighted scatter plot smoothing (LOWESS) (7) curve was fit to get the initial shape of $p(n)$. Likewise, an exponential function was fit to all the *p-values* obtained in each fold of the Monte Carlo cross validation (before averaging). Both the LOWESS and exponential curves are shown in (a), (b) and (c). The quotient between each LOWESS curve and its differential is shown in (d), (e) and (f). The constant quotients and the accurate exponential fits show empirically that the $p(n)$ functions show an exponential behavior.

Variable	Cell body size								
γ	$2.5 \cdot 10^{-6}$		$5 \cdot 10^{-6}$		$5 \cdot 10^{-5}$		$5 \cdot 10^{-4}$		
Comparison	$\Theta_{\alpha,\gamma}$	$\delta_{\alpha,\gamma}$	$\Theta_{\alpha,\gamma}$	$\delta_{\alpha,\gamma}$	$\Theta_{\alpha,\gamma}$	$\delta_{\alpha,\gamma}$	$\Theta_{\alpha,\gamma}$	$\delta_{\alpha,\gamma}$	$\hat{n}_\alpha$
Control - 1 nM Taxol	1	8.67	0	-4.12	0	-41.26	-	-	670
Control - 50 nM Taxol	1	9.37	1	4.82	0	-9.69	-	-	250
1 nM - 50 nM Taxol	1	5.38	1	3.74	0	-1.47	0	5.84	83
Variable	Cell body perimeter								
γ	$2.5 \cdot 10^{-6}$		$5 \cdot 10^{-6}$		$5 \cdot 10^{-5}$		$5 \cdot 10^{-4}$		
Comparison	$\Theta_{\alpha,\gamma}$	$\delta_{\alpha,\gamma}$	$\Theta_{\alpha,\gamma}$	$\delta_{\alpha,\gamma}$	$\Theta_{\alpha,\gamma}$	$\delta_{\alpha,\gamma}$	$\Theta_{\alpha,\gamma}$	$\delta_{\alpha,\gamma}$	$\hat{n}_\alpha$
Control - 1 nM Taxol	1	1.37	0	-17.84	0	-69.41	-	-	1160
Control - 50 nM Taxol	0	-5.75	0	-29.94	0	-90.28	-	-	1331
1 nM - 50 nM Taxol	1	9.44	1	4.69	0	-10.43	-	-	257
Variable	Cell body roundness								
γ	$2.5 \cdot 10^{-6}$		$5 \cdot 10^{-6}$		$5 \cdot 10^{-5}$		$5 \cdot 10^{-4}$		
Comparison	$\Theta_{\alpha,\gamma}$	$\delta_{\alpha,\gamma}$	$\Theta_{\alpha,\gamma}$	$\delta_{\alpha,\gamma}$	$\Theta_{\alpha,\gamma}$	$\delta_{\alpha,\gamma}$	$\Theta_{\alpha,\gamma}$	$\delta_{\alpha,\gamma}$	$\hat{n}_\alpha$
Control - 1 nM Taxol	1	9.17	0	-2.67	0	-37.41	-	-	617
Control - 50 nM Taxol	1	3.42	1	2.57	0	-0.25	0	-2.87	47
1 nM - 50 nM Taxol	1	2.39	1	1.84	1	0.05	0	-1.59	29
Variable	Protrusions binary								
γ	$2.5 \cdot 10^{-6}$		$5 \cdot 10^{-6}$		$5 \cdot 10^{-5}$		$5 \cdot 10^{-4}$		
Comparison	$\Theta_{\alpha,\gamma}$	$\delta_{\alpha,\gamma}$	$\Theta_{\alpha,\gamma}$	$\delta_{\alpha,\gamma}$	$\Theta_{\alpha,\gamma}$	$\delta_{\alpha,\gamma}$	$\Theta_{\alpha,\gamma}$	$\delta_{\alpha,\gamma}$	$\hat{n}_\alpha$
Control - 1 nM Taxol	0	0.00	0	0.00	0	0.00	-	-	-
Control - 50 nM Taxol	1	5.72	1	4.72	1	1.41	0	-1.55	42
1 nM - 50 nM Taxol	1	5.72	1	4.72	1	1.46	0	-1.42	41
Variable	Protrusions size								
γ	$2.5 \cdot 10^{-6}$		$5 \cdot 10^{-6}$		$5 \cdot 10^{-5}$		$5 \cdot 10^{-4}$		
Comparison	$\Theta_{\alpha,\gamma}$	$\delta_{\alpha,\gamma}$	$\Theta_{\alpha,\gamma}$	$\delta_{\alpha,\gamma}$	$\Theta_{\alpha,\gamma}$	$\delta_{\alpha,\gamma}$	$\Theta_{\alpha,\gamma}$	$\delta_{\alpha,\gamma}$	$\hat{n}_\alpha$
Control - 1 nM Taxol	1	11.99	1	1.15	0	-31.16	-	-	563
Control - 50 nM Taxol	1	6.23	1	4.68	0	-0.42	0	-4.73	75
1 nM - 50 nM Taxol	1	9.50	1	6.07	0	-4.98	0	-11.98	170
Variable	Protrusions perimeter								
γ	$2.5 \cdot 10^{-6}$		$5 \cdot 10^{-6}$		$5 \cdot 10^{-5}$		$5 \cdot 10^{-4}$		
Comparison	$\Theta_{\alpha,\gamma}$	$\delta_{\alpha,\gamma}$	$\Theta_{\alpha,\gamma}$	$\delta_{\alpha,\gamma}$	$\Theta_{\alpha,\gamma}$	$\delta_{\alpha,\gamma}$	$\Theta_{\alpha,\gamma}$	$\delta_{\alpha,\gamma}$	$\hat{n}_\alpha$
Control - 1 nM Taxol	1	7.75	0	-9.85	0	-58.09	-	-	754
Control - 50 nM Taxol	1	5.54	1	4.29	1	0.15	0	-3.40	58
1 nM - 50 nM Taxol	1	6.77	1	4.77	0	-1.63	0	-6.71	98
Variable	Protrusions length								
γ	$2.5 \cdot 10^{-6}$		$5 \cdot 10^{-6}$		$5 \cdot 10^{-5}$		$5 \cdot 10^{-4}$		
Comparison	$\Theta_{\alpha,\gamma}$	$\delta_{\alpha,\gamma}$	$\Theta_{\alpha,\gamma}$	$\delta_{\alpha,\gamma}$	$\Theta_{\alpha,\gamma}$	$\delta_{\alpha,\gamma}$	$\Theta_{\alpha,\gamma}$	$\delta_{\alpha,\gamma}$	$\hat{n}_\alpha$
Control - 1 nM Taxol	0	-14.70	0	-45.38	0	-114.17	-	-	1695
Control - 50 nM Taxol	1	5.10	1	3.90	0	-0.04	0	-3.45	58
1 nM - 50 nM Taxol	1	6.09	1	4.45	0	-0.90	0	-5.37	80
Variable	Protrusions diameter								
γ	$2.5 \cdot 10^{-6}$		$5 \cdot 10^{-6}$		$5 \cdot 10^{-5}$		$5 \cdot 10^{-4}$		
Comparison	$\Theta_{\alpha,\gamma}$	$\delta_{\alpha,\gamma}$	$\Theta_{\alpha,\gamma}$	$\delta_{\alpha,\gamma}$	$\Theta_{\alpha,\gamma}$	$\delta_{\alpha,\gamma}$	$\Theta_{\alpha,\gamma}$	$\delta_{\alpha,\gamma}$	$\hat{n}_\alpha$
Control - 1 nM Taxol	1	9.64	0	-4.80	0	-45.90	0	-12.24	707
Control - 50 nM Taxol	1	5.63	1	4.29	0	-0.29	0	-4.20	68
1 nM - 50 nM Taxol	1	8.34	1	5.81	0	-2.49	0	-8.57	127

Table S9. Table of decision index $\Theta_{\alpha,\gamma}$, difference $\delta_{\alpha,\gamma} = A_{\alpha_\gamma} - A_{p(n_\gamma)}$ and estimated minimum size $\hat{n}_\alpha$ for $\alpha = 0.05$ and $\gamma = 2.5 \cdot 10^{-6}, 5 \cdot 10^{-6}, 5 \cdot 10^{-5}, 5 \cdot 10^{-4}$.

Variables		Cell body size				Cell body perimeter				Cell body roundness				Protrusions binary			
		$\mathcal{N}$ grid size				$\mathcal{N}$ grid size				$\mathcal{N}$ grid size				$\mathcal{N}$ grid size			
Comparison	reduction	10	20	50	100	10	20	50	100	10	20	50	100	10	20	50	100
Control - 1 nM Taxol	0.01	80	98	82	94	95	100	99	100	71	92	71	79	100	100	100	100
	0.02	86	99	79	99	99	100	100	100	71	98	84	95	100	100	100	100
	0.1	100	100	99	100	100	100	100	100	93	100	98	100	100	100	100	100
	0.2	100	100	100	100	100	100	100	100	96	100	100	100	100	100	100	100
	1/3	100	100	100	100	100	100	100	100	100	100	100	100	100	100	100	100
	0.5	100	100	100	100	100	100	100	100	100	100	100	100	100	100	100	100
Control - 50 nM Taxol	0.01	93	100	99	100	100	100	100	100	89	100	93	94	92	99	88	87
	0.02	100	100	100	100	100	100	100	100	100	100	96	100	94	99	87	98
	0.1	100	100	100	100	100	100	100	100	100	100	100	100	100	100	100	100
	0.2	100	100	100	100	100	100	100	100	100	100	100	100	100	100	100	100
	1/3	100	100	100	100	100	100	100	100	100	100	100	100	100	100	100	100
	0.5	100	100	100	100	100	100	100	100	100	100	100	100	100	100	100	100
1 nM - 50 nM Taxol	0.01	95	99	98	98	98	100	100	100	80	94	81	89	79	98	78	91
	0.02	98	100	100	100	99	100	100	100	89	99	93	96	90	99	90	99
	0.1	100	100	100	100	100	100	100	100	100	100	100	100	100	100	98	100
	0.2	100	100	100	100	100	100	100	100	100	100	100	100	100	100	100	100
	1/3	100	100	100	100	100	100	100	100	100	100	100	100	100	100	100	100
	0.5	100	100	100	100	100	100	100	100	100	100	100	100	100	100	100	100

Variables		Protrusions size				Protrusions perimeter				Protrusions length				Protrusions diameter			
		n grid size				n grid size				n grid size				n grid size			
Comparison	reduction	10	20	50	100	10	20	50	100	10	20	50		10	20	50	100
Control - 1 nM Taxol	0.01	45	28	46	51	76	79	80	82	88	84	97	98	76	84	76	77
	0.02	36	35	50	60	74	65	79	90	86	89	99	100	78	79	85	90
	0.1	51	36	61	71	78	58	98	100	98	98	100	100	86	82	97	99
	0.2	64	24	59	83	89	57	100	100	99	100	100	100	91	95	100	100
	1/3	68	22	75	97	97	68	100	100	100	100	100	100	98	98	100	100
	0.5	66	17	82	99	98	75	100	100	100	100	100	100	100	100	100	100
Control - 50 nM Taxol	0.01	76	79	91	95	82	73	84	99	74	69	88	93	74	67	90	97
	0.02	91	98	99	99	85	85	95	98	75	89	93	98	83	86	94	98
	0.1	100	100	100	100	100	100	100	100	100	100	100	100	100	100	100	100
	0.2	100	100	100	100	100	100	100	100	100	100	100	100	100	100	100	100
	1/3	100	100	100	100	100	100	100	100	100	100	100	100	100	100	100	100
	0.5	100	100	100	100	100	100	100	100	100	100	100	100	100	100	100	100
1 nM - 50 nM Taxol	0.01	71	67	96	99	73	67	92	100	72	75	90	95	75	72	94	100
	0.02	92	80	100	100	87	84	99	100	94	90	98	100	86	87	99	100
	0.1	100	100	100	100	98	100	100	100	99	100	100	100	100	100	100	100
	0.2	100	100	100	100	100	100	100	100	100	100	100	100	100	100	100	100
	1/3	100	100	100	100	100	100	100	100	100	100	100	100	100	100	100	100
	0.5	100	100	100	100	100	100	100	100	100	100	100	100	100	100	100	100

Table S10. Table of results for different sizes of $\mathcal{N}$ and $\mathcal{F}$ grids. Each value represents the probability (%) of obtaining the same decision index $\Theta_{0.05,5\cdot10^{-06}}$ as the one shown in Table S9.
